# Identification of targetable vulnerabilities of PLK1-overexpressing cancers by synthetic dosage lethality

**DOI:** 10.1016/j.xgen.2025.100876

**Published:** 2025-05-09

**Authors:** Chelsea E. Cunningham, Frederick S. Vizeacoumar, Yue Zhang, Liliia Kyrylenko, Simon Both, Vincent Maranda, He Dong, Jared D.W. Price, Peng Gao, Konrad Wagner, Yingwen Wu, Mary Lazell-Wright, Ashtalakshmi Ganapathysamy, Rithik Hari, Kalpana K. Bhanumathy, Connor Denomy, Anjali Saxena, Jeff P. Vizeacoumar, Alain Morejon Morales, Faizaan Khan, Shayla Mosley, Angie Chen, Tetiana Katrii, Ben G.E. Zoller, Karthic Rajamanickam, Prachi Walke, Lihui Gong, Hardikkumar Patel, Hussain Elhasasna, Renuka Dahiya, Omar Abuhussein, Anton Dmitriev, Tanya Freywald, Erika Prando Munhoz, Eytan Ruppin, Joo Sang Lee, Katharina Rox, Martin Koebel, Laura Hopkins, Cheng Han Lee, Sunil Yadav, Gilles Gasparoni, Jörn Walter, Anand Krishnan, Raju Datla, Behzad Toosi, Kristi Baker, Jalna Meens, David W. Cescon, Laurie Ailles, Scot C. Leary, Yuliang Wu, Martin Empting, Alexandra K. Kiemer, Andrew Freywald, Franco J. Vizeacoumar

**Affiliations:** 1 Department of Oncology, College of Medicine, University of Saskatchewan, Saskatoon, SK S7N 5E5, Canada; 2 Department of Pathology and Laboratory Medicine, University of Saskatchewan, Saskatoon, SK S7N 5E5, Canada; 3 Department of Pharmacy, Pharmaceutical Biology, Saarland University, PharmaScienceHub, 66123 Saarbrücken, Germany; 4 Global Institute for Food Security, University of Saskatchewan, Saskatoon, SK S7N 4L8, Canada; 5 Agriculture and Agri-Food Canada, Saskatoon Research and Development Centre, 107 Science Place, Saskatoon, SK S7N 0X2, Canada; 6 Antiviral & Antivirulence Drugs (AVID), Helmholtz Institute for Pharmaceutical Research, Saarland (HIPS), Helmholtz Centre for Infection Research (HZI) and Department of Pharmacy, Saarland University, 66123 Saarbrücken, Germany; 7 Department of Oncology, University of Alberta, Edmonton, AB T6G 1Z2, Canada; 8 Department of Anatomy, Physiology, and Pharmacology, University of Saskatchewan, and Cameco MS Neuroscience Research Centre, 701 Queen St., Saskatoon, SK S7K 0M7, Canada; 9 Cancer Data Science Laboratory, National Cancer Institute, National Institutes of Health, Bethesda, MD 20892, USA; 10 Center for Bioinformatics and Computational Biology and Department of Computer Sciences, University of Maryland, College Park, MD 20742, USA; 11 Department of Precision Medicine, School of Medicine and Department of Artificial Intelligence, Sungkyunkwan University, Suwon 16419, Republic of Korea; 12 Department of Chemical Biology (CBIO), Helmholtz Center for Infection Research (HZI), Inhoffenstrasse 7, 38124 Braunschweig, Germany; 13 Department of Pathology, University of Calgary, Calgary, AB, Canada; 14 Department of Laboratory Medicine and Pathology, University of Alberta, Edmonton, AB, Canada; 15 Department of Genetics, Saarland University, Saarbrücken, Germany; 16 Western College of Veterinary Medicine, University of Saskatchewan, Room 2343, 52 Campus Drive, Saskatoon S7N 5B4, Canada; 17 Princess Margaret Cancer Centre, University Health Network, Toronto, ON, Canada; 18 Department of Biochemistry, Microbiology, and Immunology, University of Saskatchewan, Saskatoon, SK S7N 5E5, Canada; 19 Center for Gender-Specific Biology and Medicine (CGBM), 66421 Homburg, Germany; 20 Cancer Research, Saskatchewan Cancer Agency, 107 Wiggins Road, Saskatoon, SK S7N 5E5, Canada

**Keywords:** synthetic dosage lethality, tumor heterogeneity, PLK1, chromosomal instability, perturb-seq, single-cell CRISPR screening, *in vivo* CRISPR screen, IGF2BP2, IMP2

## Abstract

Chromosomal instability (CIN) drives tumor heterogeneity, complicating cancer therapy. Although Polo-like kinase 1 (PLK1) overexpression induces CIN, direct inhibition of PLK1 has shown limited clinical benefits. We therefore performed a genome-wide synthetic dosage lethality (SDL) screen to identify effective alternative targets and validated over 100 candidates using *in vivo* and *in vitro* secondary CRISPR screens. We employed direct-capture Perturb-seq to assess the transcriptional consequences and viability of each SDL perturbation at a single-cell resolution. This revealed IGF2BP2 as a critical genetic dependency that, when targeted, downregulated PLK1 and significantly restricted tumor growth. Mechanistic analyses showed that IGF2BP2 loss disrupted cellular energy metabolism and mitochondrial ATP production by downregulating PLK1 levels as well as genes associated with oxidative phosphorylation. Consistent with this, pharmacological inhibition of IGF2BP2 severely impacts the viability of PLK1-overexpressing cancer cells addicted to higher metabolic rates. Our work offers a novel therapeutic strategy against PLK1-driven heterogeneous malignancies.

## Highlights

• Synthetic dosage lethality of PLK1 can overcome tumor heterogeneity
• IGF2BP2 loss disrupts PLK1 expression and mitochondrial metabolism in cancer cells
• Pharmacological inhibition of IGF2BP2 preferentially kills PLK1-overexpressing tumors

## Graphical abstract

**Figure:**
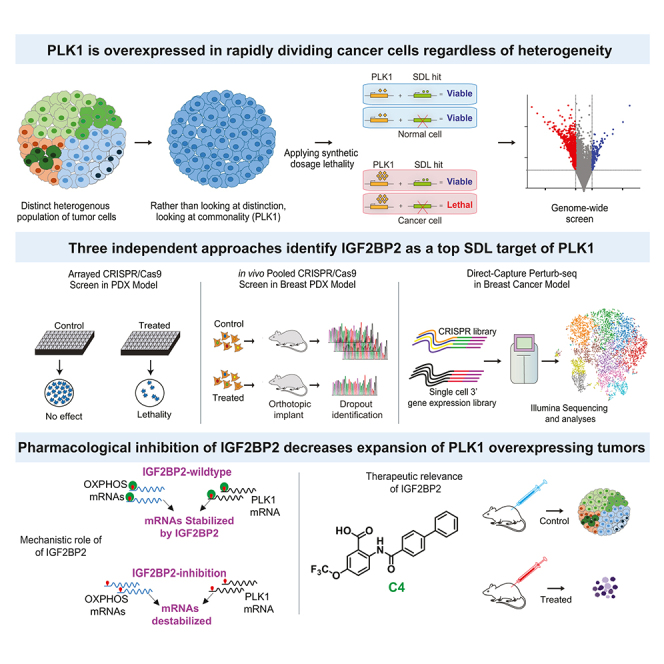

## Teaser

Direct inhibition of PLK1 has limited clinical success. Synthetic dosage lethality screening identifies IGF2BP2 as a critical genetic dependency of PLK1-overexpressing tumors. IGF2BP2 loss reduces PLK1 levels and disrupts mitochondrial metabolic functions. The pharmacological inhibition of IGF2BP2 suppresses tumor growth, offering a promising therapeutic strategy for PLK1-driven malignancies.

## Introduction

Tumor heterogeneity is an enormous clinical challenge because it provides selective, evolutionary advantages to cancer cell subsets, leading to the establishment of aggressive clones that are metastatic and treatment resistant.^1^^,^^2^^,^^3^ Current drug development programs focus on co-targeting multiple pathways within cancer cells. For example, the simultaneous co-inhibition of MEK and EGFR kinases overcomes clonal heterogeneity in colorectal cancer.^4^ Another promising approach to overcoming intratumoral heterogeneity is to target the underlying factors that drive genetic diversity, particularly chromosomal instability (CIN).^5^ This approach limits the acquisition of multi-drug resistance and treatment failure.

CIN is one of the key driving forces of genetic diversity within tumors and remains a key underlying feature of genetically diverse malignancies.^5^^,^^6^ CIN arises from aberrant mitotic division, defective double-strand break repair, replication stress, or ineffective telomere maintenance.^5^ The Polo-like kinase 1 (PLK1) is a serine/threonine protein kinase that plays a central role in controlling CIN.^7^ At the molecular level, PLK1 contributes to genome stability by signaling the initiation of mitosis, centrosome maturation, bipolar spindle formation, chromosome segregation, and cytokinesis.^7^^,^^8^^,^^9^^,^^10^^,^^11^ Constitutive overexpression of PLK1 leads to CIN and aneuploidy, which are salient features of most cancers.^12^^,^^13^ Thus, tumor cells upregulate genes such as PLK1 to support their survival and propagation.^14^^,^^15^^,^^16^^,^^17^^,^^18^ We previously demonstrated that changes in the expression of PLK1 and other genes that regulate CIN, along with factors that remodel the tumor microenvironment, represent some of the earliest events in tumor evolution.^6^ Consistent with this idea, PLK1 is overexpressed in a wide range of cancers including breast,^19^ colon,^20^ pancreatic,^21^ glioma,^22^ lung,^23^ and prostate^24^ cancers. However, 30 years after its discovery^25^ and 20 years after its pro-malignant properties were recognized,^26^ the clinical application of PLK1 inhibitors remains extremely challenging. The lack of efficacy of PLK1 inhibitors is at least in part due to the difficulty of achieving PLK1-specific inhibition.^9^ Unwanted inhibition of closely related members of the Polo-like kinase family can lead to toxicity in the nervous system^27^ or interfere with the hypoxic response and promote angiogenesis.^28^ Additionally, PLK1 plays a key role in controlling a multitude of cellular processes that regulate CIN. The complete loss of function of this protein therefore could be detrimental to normal cells and may overcome the tumor-selective basis of treatment.^9^^,^^29^ Considering the diverse functions of PLK1 and its other family members, there are still major challenges in using direct PLK1 inhibitors for cancer therapy and, as such, alternative strategies are needed.

**Figure 1:**
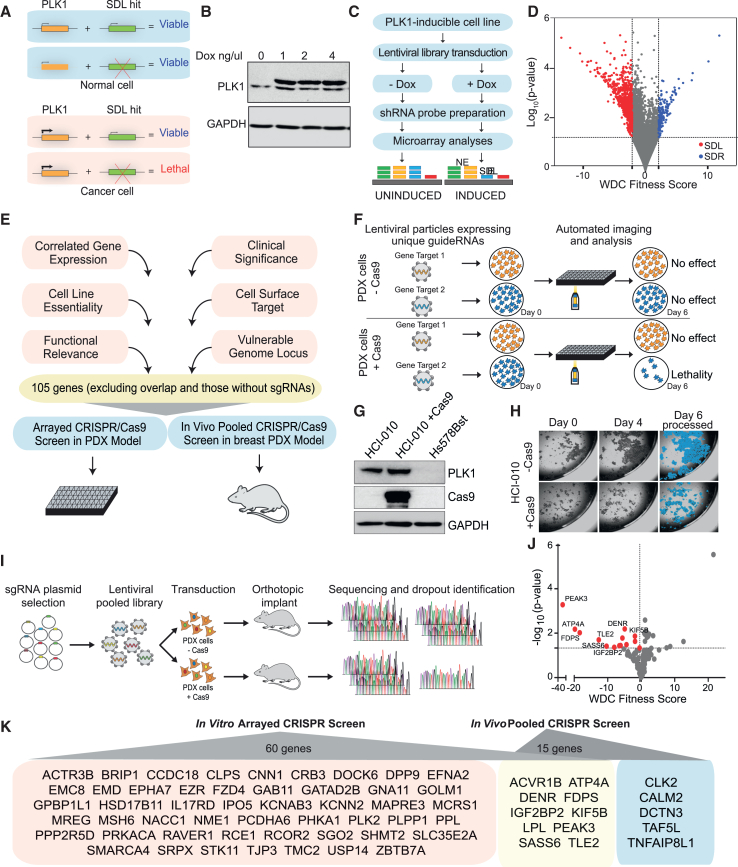
Genome-wide shRNA screening identifies synthetic dosage lethal partners of PLK1 (A) Schematic showing expected outcomes in PLK1-overexpressing cancer cells following PLK1 or PLK1-SDL gene inhibition. PLK1 inhibition may cause aneuploidy and increased cell death, while PLK1-SDL inhibition is expected to cause cell death alone. (B) Western blot of HCT116-PLK1-inducible cells with/without doxycycline. The upper band indicates phosphorylated PLK1-S137D; GAPDH serves as a loading control. (C) Schematic of genome-wide screening workflow with example microarray outcomes for non-essential (NE, green/yellow), SDL (blue), and essential (E, red) genes. (D) Volcano plot of pooled genome-wide shRNA screen. Genes significantly reducing fitness in PLK1-overexpressing vs. non-overexpressing cells (*p* < 0.05, WDC <2-fold) are shown in red (SDL hits), positives in blue. A total of 960 SDL hits were identified (full list in Table S1). (E) Schematic summarizing prioritization strategy for experimental validation. (F) CRISPR arrayed *in vitro* screening workflow in PDX breast cancer cells (±Cas9) using high-throughput imaging to assess lethality. (G) Western blot of PLK1 and Cas9 in HCI-010, HCI-010+Cas9, and non-malignant Hs578Bst cells; GAPDH used as loading control. (H) Sample images from automated imaging of different PLK1-SDL sgRNAs in −Cas9 and +Cas9 HCI-010 cells, with MetaXpress object masking (blue) shown for day 6. (I) Schematic of *in vivo* pooled CRISPR screen in PDX breast cancer model (±Cas9), with sequencing to identify sgRNA dropout. (J) Volcano plot of log_10_*p* value vs. WDC fitness score from the *in vivo* screen. Genes with *p* < 0.05 (WDC permutation-based) are significant; essential genes highlighted in red. (K) Final list of SDL hits identified across *in vitro* arrayed and *in vivo* pooled CRISPR screens.

To take advantage of frequent PLK1 overexpression in tumors, we applied a genetic approach known as synthetic dosage lethality (SDL) where the overexpression of a gene like PLK1 is only lethal when another normally non-lethal, genetic alteration is also present^30^^,^^31^ (Figure 1A). Since tumor cells upregulate genes such as PLK1, identifying SDL interactions is valuable for identifying new therapeutic targets for cancer treatment.^32^^,^^33^^,^^34^ Surprisingly, SDL remains a largely untapped area in cancer research. Here, we report on the integration of multiple unbiased platforms, including genome-wide pooled short hairpin RNA (shRNA) screening, with subsequent validation of SDL targets using pooled *in vivo* and arrayed *in vitro* CRISPR-Cas9 screens in a patient-derived xenograft (PDX) model. As PLK1 overexpression leads to cellular heterogeneity, it is also necessary to confirm whether the suppression of its SDL partner(s) truly eliminates PLK1-overexpressing cells. Therefore, we evaluated the effect of the top SDL targets at the single-cell level using a single-cell CRISPR-Cas9 screen (Perturb-seq) with direct capturing of guide RNAs.^35^ This work identified IGF2BP2 as a new potential therapeutic target and novel regulator of PLK1 expression. Subsequent studies found that IGF2BP2 loss disrupts energy metabolism and ATP production by downregulating PLK1 and oxidative phosphorylation genes. Notably, a newly characterized pharmacological inhibitor of IGF2BP2 selectively eliminated PLK1-overexpressing cells and tumors, highlighting its potential as a promising therapeutic strategy.

## Results

### Unbiased genome-wide screening revealed novel SDL interactions of PLK1

CIN is a hallmark of cancer^36^ and PLK1 overexpression has been shown to induce this response.^12^^,^^18^ To identify SDL interactions of PLK1 and exploit them in cancer therapeutics, we selected an inducible system^37^ for expressing a constitutively active PLK1-S137D mutant in HCT116 cells (HCT116-PLK1, Figure 1B). HCT116 cells harbor an intact DNA damage checkpoint and spindle assembly checkpoint with a near-diploid genome.^38^^,^^39^ Thus, this system is commonly used to study genome instability, as it does not inherently exhibit any CIN.^13^^,^^39^^,^^40^^,^^41^^,^^42^^,^^43^^,^^44^^,^^45^^,^^46^ With doxycycline-inducible expression of a PLK1-S137D mutant, we and others have shown that CIN and defects in spindle assembly checkpoint can be triggered within this model.^13^^,^^47^ Thus, unlike most breast cancer cells that have extensive aneuploidy, these PLK1-overexpressing cells serve as an excellent model system for inducing genome instability and subsequently identifying related SDL interactions.

To identify the gene knockdowns that cause lethality only when PLK1 is overexpressed, HCT116-PLK1-S137D cells that were previously shown by our team and others to induce CIN^13^^,^^47^ were transduced with a pooled lentiviral library of 90,000 shRNA sequences targeting ∼18,000 different genes, with approximately five independent hairpins per gene. Library transduction was performed at a scale of ∼300-fold representation in two distinct populations (induced vs. uninduced), as previously described^48^ (Figure 1C), and 960 gene hits were identified (Figure 1D) (with at least a 2-fold decrease and *p* < 0.05). The complete list of 960 significant hits, their corresponding weighted differential cumulative change (WDC) scores, and their biological functions are detailed in Table S1. These genes are hereafter referred to as “PLK1-SDL” hits. Although the replicates of the screen showed a high correlation (Pearson correlation coefficient, r > 0.9 between replicates) (Figure S1A), we compared our screening results with sets of essential and non-essential genes using a previously published framework to ensure that our screening reliably identified true SDL hits.^49^ This approach measured good performance by calculating the accuracy score from precision and recall tests (F-measures >0.75) (Figure S1B). The cumulative signal used to calculate the fitness score of all PLK1-SDL gene hits displayed a significant 2.2-fold decrease in the induced population from T0 to T16 (Kolmogorov-Smirnov test, *p* < 0.0001), when compared with the uninduced population from T0 to T16, which had a 1.3-fold decrease (Kolmogorov-Smirnov test, *p* < 0.0001) (Figure S1C). Consistent with this, the overlay of all individual signals from all dropouts was highly represented in the induced population compared with the uninduced population (Figure S1D). To illustrate the dropout of signals over time in the induced population alone, we analyzed one of the top hits, PPP2R5D, a regulatory subunit of the protein phosphatase 2A (PP2A) complex that we previously reported to exhibit SDL with PLK1^13^ (Figure S1E), reiterating the confidence in our screening approach. SDL interactions are functionally coherent^30^ and, as expected, upon a comparison of our PLK1-SDL hits with previously published mitosis-related screens,^50^^,^^51^^,^^52^^,^^53^^,^^54^^,^^55^^,^^56^^,^^57^ we found several hits associated with mitosis, DNA repair, and cell cycle-related pathways in addition to several completely novel PLK1 partners (Figure S1F and Table S2). Consistent with these findings, Reactome pathway analyses indicated that PLK1-SDL hits were enriched in the cell cycle (adj. *p* 1e−03), mitosis- and checkpoint-related pathways (adj. *p* < 6e−03), and RNA metabolism (adj. *p* 1e−03) and metabolic pathways (adj. *p* 1e−03) (Figure S1G). To gain insight into the functional relevance of SDL hits, literature analyses of the 960 genes were performed using Cytoscape STRING analyses. While these studies categorized SDL hits into multiple biological functions, including cell cycle progression, centrosome amplification and cytokinetic component enrichment, the extensive interactions among these components indicated that most of the SDL interactions were also functionally related (Figure S2A; Table S1).

To further increase our confidence in the enrichment analyses findings, we also used drug response data from the Genomics of Drug Sensitivity in Cancer (GDSC) repository (https://www.cancerrxgene.org) since several of the SDL hits had existing chemical inhibitors. This analysis revealed a few SDL hits with inhibitors that selectively suppressed PLK1-overexpressing cells (Figure S2B). In fact, our screening revealed that the GSK3A inhibitor CHIR-99021 preferentially affected PLK1-overexpressing cells. Similarly, the ALK receptor tyrosine kinase was identified among the SDL hits, and the ALK inhibitor NVP-TAE684 appeared to selectively target PLK1-overexpressing cells (Figure S2B). Given that ALK inhibition activates the spindle assembly checkpoint causing mitotic delay,^58^ and that PLK1-overexpressing cells may be defective in the spindle assembly checkpoint,^47^ we speculate that ALK inhibition in PLK1-overexpressing cells may lead to mitotic catastrophe. These completely independent cross-validations by chemical genetics confirmed the quality of our screens, and their potential to identify novel targets of therapeutic relevance.

### Systematic prioritization of PLK1-SDL hits for further validation studies

Genome-wide screens tend to produce false positive hits that can confound SDL identity. Most genome-wide studies cherry-pick one or two hits to serve as validated proofs of principle, but most of the data often remain unvalidated and are therefore underutilized by the research community. However, validating all individual hits from a large-scale screen, such as the one conducted here, is also practically challenging. Therefore, we applied several distinct strategies that allowed us to prioritize a larger subset of PLK1-SDL hits for experimental validation, while also maintaining the strength of a large-scale, unbiased screening approach.

Our first strategy to prioritize PLK1-SDL hits was based on the rationale that SDL genes that are differentially upregulated when PLK1 is overexpressed represent co-regulated mechanisms that become essential within the molecular context of elevated PLK1 levels.^59^^,^^60^^,^^61^ Therefore, we used patient data from The Cancer Genome Atlas (TCGA) (https://portal.gdc.cancer.gov/) to determine the number of PLK1-SDL hits that were co-upregulated with PLK1 across 33 different cancer types. We found that the expression of a subset of PLK1-SDL hits was positively correlated with PLK1 levels across several cancer types, suggesting that these hits may represent specific genetic dependencies of PLK1-overexpressing cells (Figure S3A). After filtering out essential genes^62^^,^^63^ and genes that were unlikely to be expressed in HCT116 cells (based on CCLE data^64^; log2 expression score <5.0), we selected 20 genes whose expression strongly correlated with PLK1 levels (Spearman rank correlation coefficient, r ≥ 0.4 in a majority of cancer types) (Figure S3A). In our second strategy, we investigated how many of the 960 PLK1 SDL hits were “pre-validated” in independently published essentiality screens.^62^^,^^65^ The cell lines from these published studies were classified into two groups: those with high PLK1 expression (top 10%) and those with low PLK1 expression (bottom 10%). We then determined whether our PLK1-SDL hits were more essential in the cell line group that naturally overexpressed PLK1. We found 30 SDL hits in the Marcotte et al. dataset (Figure S4A) and 19 hits in the Project Achilles dataset (Figure S4B) that had higher essentiality scores in PLK1-overexpressing cells (Wilcoxon rank-sum test, *p* < 0.05).^62^^,^^65^ A complete list with *p* values is provided in Table S3.

Our third approach assessed SDL interactions based on patient prognoses associated with the expression levels of screen hits.^59^^,^^60^^,^^61^ Briefly, patient samples were queried for “naturally occurring SDL interactions” depending on the expression levels of PLK1 and its SDL partner (Figure S4C). The genes for which naturally occurring SDL expression patterns were positively correlated with significantly improved patient survival (37 genes; Kaplan‒Meier log rank test, *p* < 0.05) were selected for further validation (Figure S4C; Table S4). Apart from the above three main approaches, we also included 21 genes from the short arm of chromosome 19 (19p13.2–3), as our screen identified many genes from this locus (Table S5). Given that this region has been associated with macrocephaly^66^ and that PLK1 and its centrosome functions have been previously linked to both micro- and macrocephaly,^67^^,^^68^ we expected to observe functional crosstalk between PLK1 and this vulnerable locus. We also included 25 functionally relevant PLK1-SDL hits, as determined by gene prioritization algorithms^69^ (Table S5). Finally, 30 potential cell surface SDL targets were included because they may represent targetable vulnerabilities for the development of advanced therapeutic strategies, such as antibody-based inhibitors (Table S5). After removing overlapping hits among these approaches, we selected 134 genes for further studies. Because 29 of these genes failed to clone, we focused our subsequent efforts on validating the remaining 105 genes (Figure 1E).

### Combination of *in vivo* pooled CRISPR and *in vitro* arrayed CRISPR screens in a PLK1-overexpressing breast cancer PDX-derived cell line model

Following the prioritization of 105 candidate SDL hits, we made a systematic effort to identify the best SDL hits as potential anti-tumor targets. To this end, we selected 210 single guide RNA (sgRNA) sequences targeting 105 genes and queried their relevance in PLK1-overexpressing cancer by using *in vivo* pooled and *in vitro* arrayed CRISPR screens (Figure 1E). Briefly, pooled lentiviral particles expressing two independent guide RNAs were transduced together to target each PLK1-SDL hit, and the confluency of cells was quantified (Figure 1F). We used the HCI-010 breast cancer PDX model^70^ that naturally overexpresses PLK1 compared with non-malignant Hs578Bst breast epithelial cells. HCI-010-derived cells were also engineered to stably express Cas9 for a CRISPR-based screen (Figure 1G). Following transduction, these cells were monitored over time and cell confluency in each well was determined using automated image analysis (Figure 1H) (Molecular Devices MetaXpress image analysis software). In total, silencing 60 of the 105 genes was associated with an approximately 40% or greater reduction in confluency (Student’s t test, *p* < 0.0.5) (Figures S5A and S5B; Table S6).

In parallel, we generated a pooled lentiviral library containing all 210 sgRNA sequences and transduced HCI-010 PDX-derived cells with or without Cas9 expression in a manner mimicking the genome-wide pooled screen, as previously described^71^ (Figure 1I). Following puromycin selection, sgRNA-transduced HCI-010 cells were introduced into the mammary fat pad regions of female mice with more than 4,000-fold representation per sgRNA (3 million cells injected containing 210 sgRNAs library). After 3 weeks of tumor growth, we harvested tumors, extracted genomic DNA, and sequenced it to measure sgRNA dropout in HCI-010 Cas9-expressing samples against a baseline control of HCI-010 tumors without Cas9 expression. Replicates of the screen showed a high correlation (Pearson correlation coefficient, r > 0.9) (Figure S6A) among the seven tumors in each condition. This *in vivo* pooled CRISPR screen identified 15 SDL hits (*p* < 0.05) (Figure 1J and Table S7). Overall, using these two complementary approaches, we validated 65 PLK1-SDL hits in the HCI-010 PDX model, with 10 SDL hits overlapping the *in vivo* and *in vitro* experiments (Figure 1K). Cas9 genome editing was confirmed using a cleavage detection assay (Figure S6B). While these 65 SDL hits were found to be essential for the survival of PLK1-overexpressing cells, we decided not to prioritize testing their essentiality in PLK1-low or non-malignant controls at this stage. Instead, we used a single-cell CRISPR screen to identify the most optimal SDL hits that could overcome tumor heterogeneity in the context of PLK1 overexpression.

### Shortlisting of the top candidates via Perturb-seq with direct guide RNA capture

**Figure 2:**
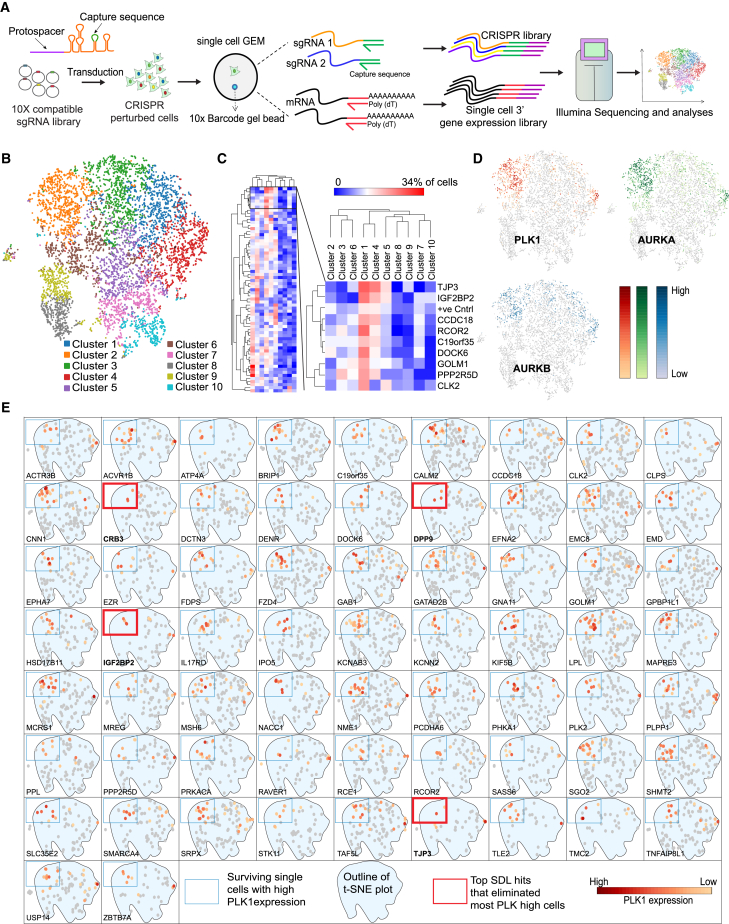
Shortlisting of the top PLK1-SDL candidates using direct guide RNA capture Perturb-seq screening (A) Schematic of Perturb-seq screening using direct guide RNA capture. A 10X-compatible guide library with capture sequence 1 in the stem loop was transduced into MCF7 cells, followed by 4 days of culture post-puromycin selection. Gene expression and CRISPR knockout (KO) libraries were prepared after cell barcoding and indexing and sequenced using NovaSeq. (B) Single-cell K-means cluster projection of the t-SNE-embedded pooled sgRNA screen showing 10 clusters (*n* = 7,434 cells). (C) Hierarchical clustering using Pearson correlation of the knockout percentage per gene across the 10 clusters. Enriched knockouts in clusters 1 and 4, along with positive controls, are presented. (D) Expression of PLK1, AURKA, and AURKB (log_2_ scale, *n* = 7,434) shows high co-expression in cluster 2. (E) Cells with high PLK1 expression (boxed cluster 2) were traced across all 65 single-gene knockouts. Knockouts of CRB3, DPP9, IGF2BP2, and TJP3 showed the fewest cells with elevated PLK1.

Given that CIN is a key contributor to tumor heterogeneity,^72^ and that PLK1 overexpression is known to induce CIN in tumor cells,^12^^,^^18^ we aimed to identify which of the 65 SDL hits we validated selectively impact the survival of PLK1-overexpressing cells at a single-cell resolution. However, bulk analyses often obscure subpopulations of cells with distinct vulnerabilities. Since tumor heterogeneity gives rise to these subpopulations, we leveraged direct-capture Perturb-seq^35^ to identify SDL dependencies most effective at eliminating PLK1-overexpressing tumor cells. We constructed a new sgRNA library targeting the 65 experimentally validated SDL genes in the Chromium Single-cell Gene Expression v3.1 LV13 vector backbone (U6-gRNA:EF1a-Puro-2a-BFP; MilliporeSigma) with 10x Genomics-compatible capture sequence 1 (CS1: 5′-TTGCTAGGACCGGCCTTAAAGC-3′) at the stem-loop position. In this system, a functionally expressed sgRNA incorporates a capture sequence directly into the guide scaffold to allow direct capture of sgRNAs during single-cell RNA sequencing.^35^ Sixteen guide sequences targeting housekeeping genes, including RPL3, RPL11, RPL13, and RPL18, were used as positive controls, as described previously,^49^ and 15 non-targeting guide sequences were used as negative controls (Table S8). This sub-library of 161 guides targeting 65 genes and the relevant controls was used to transduce MCF7 breast cancer cells, which innately overexpress PLK1^73^ (Figure 2A).

Following transduction and selection, pooled knockout cells were cultured for 4 days, and single-cell gel beads in emulsion (GEMs) with barcoded polyadenylated mRNA primers and sgRNA capture sequences were generated for single-cell transcriptome and CRISPR library construction. After sequencing and standard data processing using the 10x Genomics Cell Ranger (ver. 6.1.2) software package, the UMI matrix of cells from each replicate was extracted using single guides, subjected to quality control and normalization, and subsequently imported into the Loupe Browser (ver. 6.0) for downstream analysis and t-sne plot generation (Figure 2B). We sequenced 22,041 single cells, which yielded an average of 51,479 reads per cell, with a median of ∼4,000 genes expressed per cell. Individual cells that had fewer than 5,000 transcripts (<5,000 UMIs per cell) or were mapped to 0 or ≥2 guides in a single cell were eliminated. Overall, we considered 7,434 single cells with at least 50 representative cells for most knockouts when we mapped a single guide per cell (Figure S7A). The efficiency of each knockout was confirmed by comparing the expression of the corresponding knockout gene to that of the negative control (Figure S7B). The expression of six genes (LPL, CNN1, C19ORF35, CLPS, ATP4A, and TMC) did not decrease significantly, and these genes were excluded from further analyses (Figure S7B). The resulting t-SNE plot from the correlation of transcriptomic data across the 65 gene knockouts is outlined in 10 clusters (Figure 2B), with clusters 1 and 4 enriched for knockouts of positive controls and eight additional genes (*TJP3, IGF2BP2, CCDC18, RCOR2, DOCK6, GOLM1, PPP2R5D,* and *CLK2*) (Figure 2C). As housekeeping positive controls are expected to cause cell death, their co-enrichment upon knockout indicated that the loss of function of these eight genes aligns with the expected SDL phenotype. Moreover, the enrichment of PPP2R5D knockouts within these clusters also increases confidence in our approach, as we previously reported that PPP2R5D exhibits SDL with PLK1.^13^ Interestingly, cluster 2 was enriched in both PLK1-overexpressing and Aurora kinase-overexpressing cells (Figure 2D). Therefore, we next mapped individual gene knockouts to determine their relationship with cell populations with high PLK1 levels (Figure 2E). We found knockouts of four genes (*IGF2BP2, CRB3, DPP9,* and *TJP3*) that displayed negative selection with fewer PLK1-overexpressing cells or increased lethality in cells with higher PLK1 levels (Figure 2E). Among these, we chose IGF2BP2 as the most promising SDL candidate because it not only was picked up by direct-capture Perturb-seq but also was identified in both the *in vivo* pooled CRISPR screen and *in vitro* arrayed CRISPR screen (Figure 1K).

### Loss of IGF2BP2 affects PLK1 expression and oxidative phosphorylation

**Figure 3:**
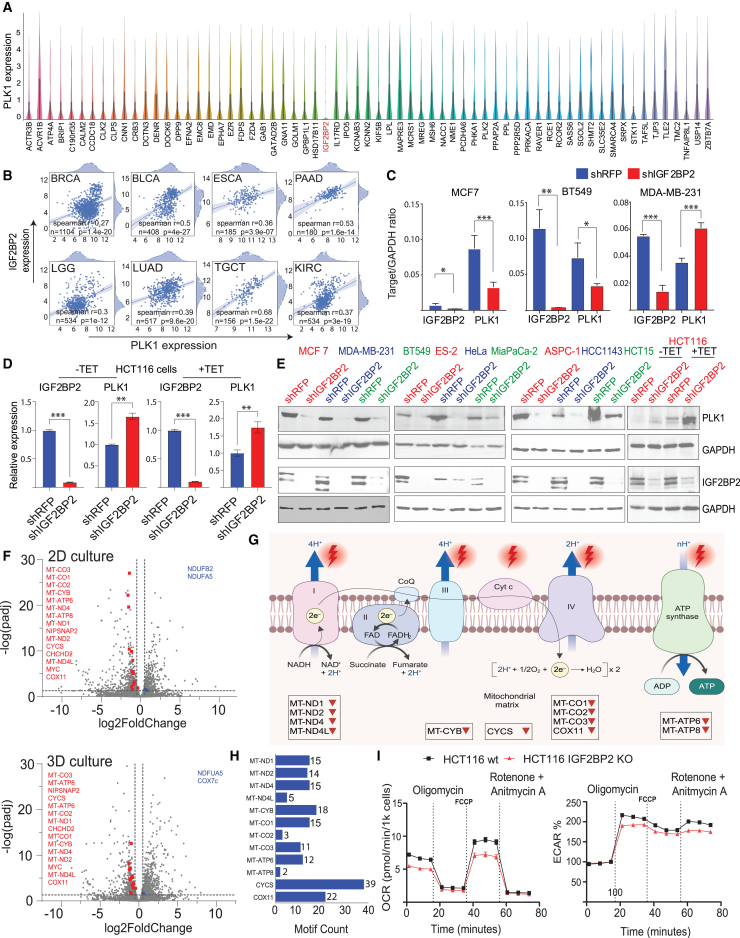
IGF2BP2 affects the expression of PLK1 and reduces cellular respiration (A) Violin plots showing PLK1 levels in pooled single cells from 65 knockouts, generated using 10x Genomics Loupe Browser. (B) PLK1 and IGF2BP2 expression were positively correlated across multiple TCGA cancer types. Spearman correlations were calculated using log_2_ RSEM-normalized RNA-seq data. Each patient is a blue dot; frequency plots along the axes indicate expression distribution. Correlation coefficient (r), sample size (*n*), and *p* values are shown; the blue line indicates best fit. (C) Digital droplet PCR results (target/GAPDH ratios) show significant PLK1 downregulation in MCF7 (*p* < 0.001) and BT549 (*p* < 0.05) cells, and upregulation in MDA-MB-231 cells (*p* < 0.001) following IGF2BP2 knockdown. (D) RT-qPCR showing IGF2BP2 and PLK1 expression in Tet-induced and uninduced HCT116 cells, relative to controls. (E) Western blot of PLK1 levels after IGF2BP2 knockdown in Tet-inducible HCT116 cells; GAPDH used as loading control. (F) Differentially expressed genes related to OXPHOS in 2D vs. 3D cultures are marked in red (downregulated) or blue (upregulated). (G) Schematic of human respiratory chain (BioRender.com), highlighting genes downregulated in HCT116 IGF2BP2 knockout cells (shared in 2D and 3D); affected complexes marked in red. *n* = 3 biological replicates. (H) Transcript sequences from Ensembl (GRCh38.p14) were analyzed using Bio.Seq v1.83. Motifs “UGGA” or “AGGU” were counted; results visualized with seaborn. (I) Mitochondrial stress tests (Agilent) were performed on HCT116 wild-type and IGF2BP2 knockout cells; OCR and ECAR values are presented.

IGF2BP2 is an *N6-methyladenosine (m6A)* reader known to bind *m6A* modifications in the 3′ untranslated region of *PLK1* mRNA, stabilizing its expression.^74^ Consistent with these findings, our direct-capture Perturb-seq analysis identified IGF2BP2 as the only target among 65 SDL hits whose loss strongly reduced *PLK1* mRNA expression (Figure 3A). In support of this observation, analysis of TCGA data revealed a positive correlation between PLK1 and IGF2BP2 expression (Spearman’s r ≥ 0.3) across multiple tumor types (Figure 3B), suggesting that IGF2BP2 modulates PLK1 levels and is thus preferentially essential in cancer cells relying on PLK1 overactivation.

To further validate this association, we performed digital droplet PCR (ddPCR) after IGF2BP2 knockdown and observed significant downregulation of *PLK1* mRNA in MCF7 (*p* < 0.001) and BT549 cells (*p* < 0.05) (Figures 3C and S8). Conversely, *PLK1* transcript levels increased in MDA-MB-231 cells (*p* < 0.001) and HCT116 cells under both doxycycline-uninduced and -induced (expressing the constitutively active PLK1-S137D mutant) conditions (Figures 3C and 3D). While *PLK1* transcript levels showed variability in MDA-MB-231 and HCT116 cells, protein abundance was consistently reduced in nine out of 10 independent cell line models, except in HCT116, suggesting that additional regulatory mechanisms are at play (Figure 3E).

To explore this possibility, transcriptomic analyses were conducted on HCT116 cells with and without IGF2BP2 in monolayer and 3D spheroid cultures. RNA sequencing revealed over 1,200 differentially expressed genes in monolayer cultures and over 1,300 in spheroids (Table S9). Notably, genes associated with the “oxidative phosphorylation” (OXPHOS) pathway were consistently downregulated in IGF2BP2-deficient cells (Figure 3F). These included mitochondrially encoded genes (e.g., NADH-ubiquinone oxidoreductase subunits and cytochrome *c* oxidase subunits) and nuclear-encoded, mitochondrial genes (e.g., cytochrome *c*) whose protein products are vital to the function of every dually encoded OXPHOS complex (Figure 3G). In support of this, *m6A* modifications are enriched in mitochondrially related RNAs, with loss of *m6A* leading to reduced respiratory capacity.^75^ As crystallographic studies of the KH34 domain of IGF2BP2 have identified AGGU and UGGA motifs as key RNA-binding regions,^76^ we investigated whether these motifs are present in the mRNAs of downregulated OXPHOS-related genes. Indeed, these analyses revealed multiple occurrences of AGGU and UGGA motifs in the mRNAs of OXPHOS-related genes (Figure 3H), indicating that IGF2BP2 can regulate the stability of mRNAs of multiple gene products from this biochemical pathway. Consistent with these findings, mitochondrial stress tests confirmed that IGF2BP2-deficient cells exhibited decreased metabolic activity and lower basal and maximal respiration rates (Figure 3I). These results suggest that IGF2BP2 loss disrupts OXPHOS and by extension mitochondrial ATP production, preferentially impairing the viability of PLK1-overexpressing cancer cells, which are known to be addicted to higher metabolic activity.^77^^,^^78^^,^^79^^,^^80^^,^^81^^,^^82^^,^^83^^,^^84^^,^^85^^,^^86^ In summary, loss of IGF2BP2 decreases the viability of PLK1-overexpressing cells by two synergistic mechanisms: first by reducing PLK1 levels and second by impairing their capacity to produce energy. Both mechanisms are potentially operative in most cancer cells, where IGF2BP2 inhibition decreases PLK1 expression.

### Loss of IGF2BP2 suppresses PLK1-overexpressing cancer cells, tumorspheres, and tumors

**Figure 4:**
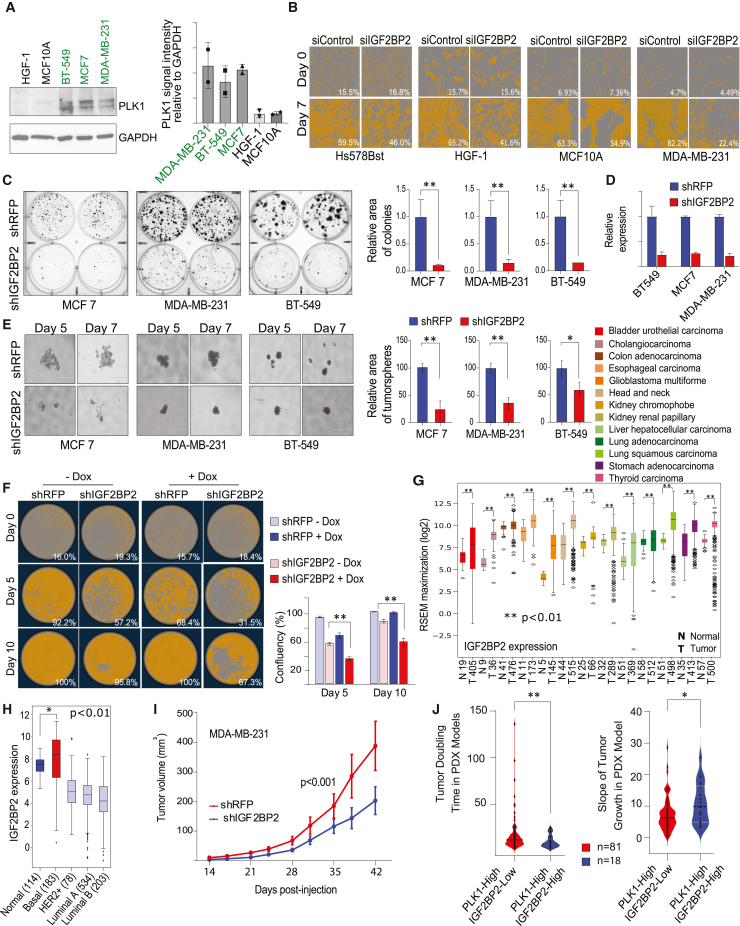
Loss of IGF2BP2 suppresses the growth of PLK1-overexpressing cells and tumors (A) Western blot showing PLK1 protein levels in non-malignant and malignant cells; quantification is shown on the right. (B) Sample images with orange cell masking (S3-IncuCyte) of non-malignant cells transfected with scrambled or IGF2BP2-targeting siRNA. Representative images from day 0 and day 7 are shown. PLK1-overexpressing MDA-MB-231 cells served as a control. Mean confluency from independent replicates (*n* = 3) is indicated in white. (C) Colony formation assay in breast cancer cells after IGF2BP2 knockdown; shRFP used as control. Colonies were quantified in ImageJ by area, in MCF7 (*p* < 0.01), MDA-MB-231 (*p* < 0.01), and BT549 (*p* < 0.05). (D) qPCR confirming IGF2BP2 knockdown following shIGF2BP2 lentiviral transduction vs. shRFP. (E) Representative tumorsphere images from MCF7, MDA-MB-231, and BT549 cells (scale bar, 1,000 μm), imaged with EVOS m5000. Tumorsphere area per well, quantified using ImageJ, was significantly reduced after IGF2BP2 knockdown (MCF7/MDA-MB-231: *p* < 0.01; BT549: *p* < 0.05). (F) Images with orange cell masking and quantification of PLK1-inducible HCT116 cells following IGF2BP2 knockdown using S3-IncuCyte. (G) TCGA data showing IGF2BP2 overexpression in multiple cancers (*p* < 0.01, Wilcoxon rank-sum test). Cancer type abbreviations follow the TCGA portal; *x* axis shows sample numbers. (H) TCGA breast cancer data classified by PAM50 shows IGF2BP2 is significantly overexpressed in TNBC. (I) Tumor volume after IGF2BP2 knockdown: MDA-MB-231 cells (2 × 10^6^) transduced with shIGF2BP2 or shRFP were injected into NSG mice (*n* = 10/group), with tumor volumes measured every 3–4 days. Tumor volume was significantly reduced in the knockdown group (*p* < 0.001, two-way ANOVA). (J) PDX models^88^ were categorized by PLK1 and IGF2BP2 expression. Tumor doubling times and growth slopes are shown for each group. Significance was determined by unpaired t test with Welch’s correction (∗*p* < 0.05; ∗∗*p* < 0.01); sample sizes are indicated.

We next investigated the role of IGF2BP2 as a therapeutic cancer target. Toward this end, we screened several non-malignant cell lines for PLK1 levels and found that Hs578Bst (Figure 1G), HGF-1, and MCF10A had limited expression of PLK1 (Figure 4A). As most non-malignant cells did not grow efficiently following lentiviral PLK1 knockdown, we transfected these cells with small interfering RNAs (siRNAs), and confluency was monitored using live-cell imaging (S3-IncuCyte). IGF2BP2 knockdown minimally affected these non-malignant cells over the 7-day monitoring period compared with the corresponding PLK1 high MDA-MB-231 and control cells transfected with non-silencing siRNA (Figure 4B). In contrast, similar siRNA transduction in the malignant MDA-MB-231 cells caused a strong growth reduction (Figure 4B). The efficiency of IGF2BP2 knockdown in all models was confirmed by qRT-PCR (Figure S9A). Furthermore, knockdown of IGF2BP2 also significantly decreased colony formation in a panel of breast cancer cell lines when compared with control shRNA-transduced cells (Figures 4C and 4D). As tumorsphere models better simulate tumor biology compared with cells cultivated in monolayers,^87^ we also investigated the effects of IGF2BP2 loss under these conditions. Our findings revealed that knockdown of IGF2BP2 also effectively suppressed the growth of breast cancer cells in tumorspheres (Figure 4E). Similar results were observed in the HCT116-PLK1-inducible model cell line that was used in the genome-wide screen, with loss of IGF2BP2 causing a preferential decrease in cell viability in the PLK1-induced condition (Figure 4F). Overall, these *in vitro* analyses support the potential for IGF2BP2 inhibition in the selective elimination of PLK1-overexpressing cancer cells.

To evaluate the translational potential of IGF2BP2 as a cancer treatment target, we examined the expression of IGF2BP2 in patient samples available in TCGA and found that it is significantly overexpressed in multiple cancers, including triple-negative breast cancer (TNBC) (Figures 4G and 4H). To expand upon these *in silico* findings, we examined whether IGF2BP2 loss reduces tumor growth in TNBC xenograft models *in vivo*. Individual shRNAs were used to silence IGF2BP2 in MDA-MB-231 cells, which were then injected into the mammary pad regions of immunodeficient female NOD.Cg-*Prkdc*^*scid*^*Il2rg*^*tm1Wjl*^/SzJ (NSG) mice. Consistent with our findings in tumorsphere models, we observed that silencing IGF2BP2 strongly reduced the growth of MDA-MB-231 tumors innately overexpressing PLK1 (Figure 4I).

To derive additional support for our findings, we took advantage of multiple previously described PDX models representing different tissue types.^88^^,^^89^ We evaluated the tumor growth patterns of 174 PDX models that were not subjected to any drug treatment and classified them into two groups based on the expression patterns of PLK1 and IGF2BP2. Group 1 had high PLK1 expression but low IGF2BP2 expression, while group 2 had both high PLK1 and IGF2BP2 expression. After analyzing the doubling time and slope of tumor growth, we found that the PDX models in group 1 had a significantly longer doubling time and a significantly flatter growth slope than those in group 2 (*p* < 0.05, unpaired t test with Welch’s correction) (Figure 4J). These results agreed with our earlier observations in cultured cancer cells and emphasized that IGF2BP2 displays an SDL interaction with PLK1 and that its silencing selectively suppresses the growth of PLK1-overexpressing tumors in PDX models.

### Pharmacological inhibition of IGF2BP2 attenuates PLK1 expression and decreases the expansion of PLK1-overexpressing cells and tumors

**Figure 5:**
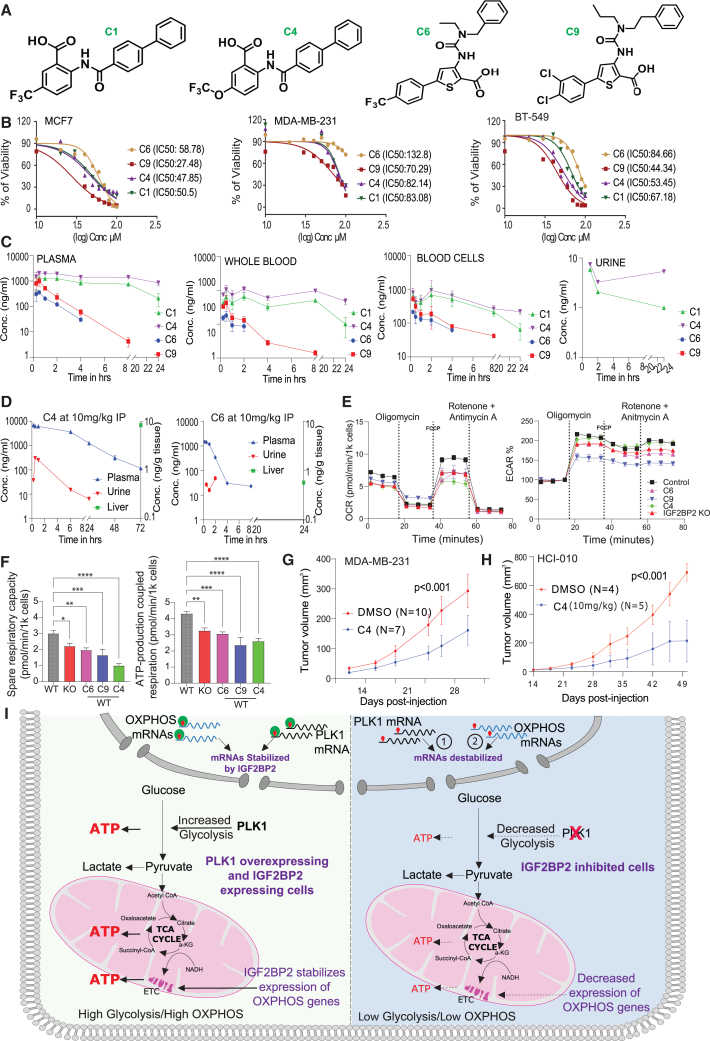
Pharmacological inhibition of IGF2BP2 reduces tumor growth (A) Chemical structures of tested IGF2BP2 inhibitors. (B) Dose-response curves for compounds C1, C4, C6, and C9 in PLK1-overexpressing cell lines. (C) Concentrations of C1, C4, C6, and C9 in plasma, whole blood, blood cells (0.25–24 h), and urine (1, 2, 24 h) after i.v. administration (1 mg/kg). (D) Pharmacokinetics of C4 and C6 after i.v. injection (10 mg/kg): plasma levels of C4 (0.25–72 h) and C6 (0.25–24 h); liver concentrations at selected time points. (E) Mitochondrial stress test (Agilent) in HCT116 wild-type and IGF2BP2 knockout cells treated with 50 μM of C4, C6, C9, or DMSO for 4 h (*n* = 6 technical replicates). OCR and ECAR compared between treated wild-type and knockout cells. (F) Spare respiratory capacity (left) and ATP-linked respiration (right) of HCT116 wild-type cells treated with IGF2BP2 inhibitors compared with knockout cells. (G and H) Effect of C4 on tumor growth: MDA-MB-231 (2 × 10^6^) or HCI-010 (1 × 10^6^) cells were injected into mammary fat pads of female NSG mice. C4 (10 mg/kg) or DMSO was delivered i.p. in 2-hydroxypropyl-β-cyclodextrin, 5 days on/2 off for 30 days. Tumor volume (±SEM) measured every 3–4 days; group size shown on graph. Significance by nonlinear regression with least squares fit. (I) Proposed model: In IGF2BP2-intact cells, glycolysis and mitochondrial metabolism cooperate to generate ATP. IGF2BP2 inhibition reduces PLK1 mRNA (1) and downregulates OXPHOS-related genes (2) leading to cell death.

Our team recently described the first small-molecule inhibitors of IGF2BP2 (Figure 5A).^90^ Therefore, we generated dose-response curves for four of these inhibitory compounds in multiple PLK1-overexpressing breast cancer cell lines to determine their half maximal inhibitory concentration (IC_50_). While lower concentrations of C4 and C9 compounds suppressed PLK1 overexpressing cancer cells more effectively (Figure 5B), compound C4 showed the best *in vivo* pharmacokinetic (PK) profile of the tested IGF2BP2 inhibitors. The highest exposures were observed in the plasma, whole blood, blood cells, and urine after intravenous (i.v.) administration in mice, with a half-life of approximately 22 h. In contrast, C6 and C9 had relatively low half-lives of only 1 h, while C1 had a half-life of approximately 9 h. Moreover, C4 displayed a high plasma concentration of approximately 1.5 μg/mL after a relatively low i.v. dose of 1 mg/kg. C4 and C1 also exhibited low volumes of distributions (∼0.5 L/kg and 0.7 L/kg, respectively) and low clearances (∼0.3 mL/min/kg and 0.9 mL/min/kg, respectively). In contrast, compounds C6 and C9 had moderate-to-low clearance rates of 25 and 10 mL/min/kg, respectively (Figure 5C). As C4 exhibited the best PK parameters of the four compounds tested in the cassette PK study, we explored the intraperitoneal (i.p.) route at 10 mg/kg to enable serial administration over several days. Moreover, we tested C6 because it had a greater distribution volume of approximately 2 L/kg after i.v. administration, and we wanted to probe the terminal compound levels in the liver (Figure 5D). C4 showed sustained plasma levels with a C_max_ of 7.1 μg/mL and a mean residence time of approximately 15 h. No accumulation was observed as the plasma levels continually decreased over 72 h. This finding suggests that a dosing interval of >24 h could be used for efficacy studies of C4. Indeed, compound C4 was also detected in the urine after 24 h. Compound C6 had a C_max_ of approximately 1.5 μg/mL with a T_max_ of 0.4 h and a mean residence time of approximately 1.9 h. Compound C6 had terminal liver levels of approximately 32 ng/g tissue after 24 h, whereas compound C4 still exhibited terminal liver levels of approximately 8.4 ng/g after 72 h. Moreover, C4 had a bioavailability of approximately 32%, whereas C6 had a bioavailability of approximately 38%, after i.p. administration.

We next explored whether the application of C4, C6, and C9 had any effect on mitochondrial activity. Treatment with these IGF2BP2 inhibitors decreased both basal and maximal respiration rates, mimicking the effect of knocking out IGF2BP2 (Figure 5E). While C9 treatment resulted in greater proton leak as the oxygen consumption rate (OCR) remained slightly higher after oligomycin treatment, C4- and C6-treated cells mirrored IGF2BP2 knockout cells in these experiments. Moreover, the calculation of spare respiratory capacity and ATP production-coupled respiration revealed additional similarities between inhibitor-treated and IGF2BP2 knockout cells (Figure 5F). Consistent with these observations, supplementing C4-treated cells with exogenous ATP partially rescued cancer cell viability while equivalent concentrations of a non-hydrolyzable ATP analog did not (Figure S9B), providing further experimental evidence in support of a direct role for metabolic disruption in the SDL action of IGF2BP2.

Based on their individual PK features and effects on ATP production, we selected C4 for further *in vitro* and *in vivo* studies. We first assessed the effect of C4 treatment on PLK1 levels and observed that PLK1 abundance was significantly decreased (Figure S9C). This C4 effect was due to the inhibition of IGF2BP2 activity, but not its downregulation, as abundance of this protein was not affected by C4 treatment (Figure S9C). Next, a Poly (ADP-ribose) polymerase (PARP) cleavage experiment was performed to determine whether apoptotic pathways were activated in cells following C4 treatment. Indeed, we observed cleaved PARP in all innately PLK1-high cancer cells treated with higher doses of the compound (Figure S9C). To further evaluate the therapeutic potential of IGF2BP2 inhibition, we examined whether C4 treatment reduced tumor development in PLK1-high xenograft and PDX models of TNBC. These experiments revealed that C4 administration suppressed tumor growth in TNBC xenografts generated with MDA-MB-231 cells (Figure 5G) and HCI-010 PDX-derived cells (Figure 5H). Taken together, these studies suggest that IGF2BP2 inhibitors have high therapeutic potential and that with further refinement the application of these compounds would allow us to effectively reduce PLK1 protein levels and ultimately suppress PLK1-overexpressing cancer cells and tumors.

## Discussion

Multiple PLK1-targeting compounds have been identified and are currently being assessed in clinical trials.^91^ The most notable trials have been performed with BI2536, BI6727 (volasertib), and GSK461364A, which are ATP-competitive inhibitors of PLK1. However, monotherapy with BI2536 was terminated due to a low objective response rate and poor survival,^92^ while clinical studies with GSK461364 were associated with a high incidence of venous thrombotic emboli.^93^ Volasterib, a derivative of BI2536, was initially successful in gaining Food and Drug Administration breakthrough therapy status but its application has yet to show significant, promising results.^94^ New inhibitors of PLK1, such as TAK960 and NMS-P937, are still in their early stages of development.^91^ These findings indicate the importance of PLK1 targeting and highlight the challenges associated with its direct inhibition.

In this study, we leveraged a systematic and unbiased genome-wide screening approach to identify genes essential for the survival of PLK1-overexpressing cells. Our primary goal was to develop a robust framework to pinpoint optimal therapeutic targets. Using an engineered model cell line, we performed an initial high-throughput screen to broadly query the genome. These results were then validated using a PDX-derived cell line and a PDX-based xenograft model that naturally overexpresses PLK1, providing translational relevance. The integration of multiple screening approaches was pivotal in refining our results. Genome-wide screening, complemented by orthogonal tools such as shRNAs and sgRNA reagents helped mitigate off-target effects and prioritize robust candidates. Importantly, single-cell CRISPR screening (Perturb-seq) enabled us to evaluate candidate gene efficiency in the context of PLK1 overexpression-induced CIN and intratumoral heterogeneity. This stepwise approach identified several overlapping genes that regulate key cancer processes, including epithelial-to-mesenchymal transition, tumor stemness, and cytokinesis. Among these, IGF2BP2 emerged as the most promising therapeutic target due to its role in maintaining tumor-initiating cell populations across multiple cancer types, including colon cancer and gliomas.^95^^,^^96^ By combining comprehensive genome-wide screening with in-depth validation, our study highlights IGF2BP2 as a compelling candidate for targeted therapy in PLK1-overexpressing cancers.

IGF2BP2 is an *m6A* reader that binds to *m6A* in the 3′ untranslated region of *PLK1* mRNA, stabilizing its expression.^74^ IGF2BP2 also stabilizes mRNAs of complexes essential for OXPHOS and, therefore, aerobic ATP generation.^96^^,^^97^ This is interesting because extensive evidence suggests that, like IGF2BP2, PLK1 is linked to metabolic regulation.^77^^,^^78^^,^^79^^,^^80^^,^^81^^,^^82^^,^^83^^,^^84^^,^^85^^,^^86^ For example, PLK1 can influence mitochondrial dynamics and bioenergetics by phosphorylating AMPKα2 to activate fatty acid oxidation and OXPHOS.^82^ As a result, PLK1-overexpressing cancer cells are heavily reliant on high rates of ATP production. Our data thus argue that targeting IGF2BP2 likely produces an SDL effect in PLK1-overexpressing cancer cells by reducing PLK1 expression and its associated activities, while simultaneously decreasing the stability of mitochondrial transcripts encoding core OXPHOS subunits; a synergistic effect that greatly reduces the capacity to generate ATP (Figure 5I). This combined disruption of the major energy-producing pathway in PLK1-overexpressing cancer cells impairs energy metabolism, which in turn severely affects their viability. Consistent with this idea, the viability of PLK1-overexpressing cancer cells in the absence of IGF2BP2 activity was partially rescued by supplying exogenous ATP in our experiments.

Our detailed pre-clinical characterization of the previously identified first small-molecule inhibitors of IGF2BP2, particularly compound C4,^90^ represents a significant advancement toward the development of novel cancer therapeutics. Compound C4 not only demonstrated superior PK properties but also effectively mimicked the metabolic impacts of IGF2BP2 deficiency, including decreased mitochondrial respiration and ATP production. These findings highlight the potential of C4 as a viable prototype for developing therapeutic compounds targeting IGF2BP2 in PLK1-overexpressing cancers. Although the concentration of C4 required to inhibit IGF2BP2 is relatively high, it is important to note that at the i.p. dose of 10 mg/kg, C4 achieved sufficient plasma concentrations to justify further efficacy studies. Nevertheless, we acknowledge that while anti-tumor effects of C4 serve as a proof of principle for the efficiency of pharmacological interference with IGF2BP2 activity for cancer treatment, C4 is not likely to serve as an actual drug for cancer therapy. We expect that C4 could potentially be used as a prototype molecule for future drug development studies. Further work will be required to optimize C4 structure to increase its efficacy, which would in turn allow for reduced therapeutic doses and greatly diminish potential off-target effects.

While we focused solely on IGF2BP2, further investigation of the other three top SDL candidates is needed to determine their relationships with PLK1. For example, CRB3 and TJP3 are involved in the establishment of tight junctions^98^^,^^99^ and may affect cytokinesis, as PLK1 also plays a key role in this process.^100^ In fact, among the pleiotropic defects caused by PLK1 overexpression,^7^^,^^8^^,^^9^^,^^10^^,^^11^ cytokinesis failure and abscission are the most common and result in aneuploidy.^12^ DPP9 is a peptidase that degrades most cytosolic proline-containing peptides^101^ and has been shown to interact specifically with SUMO1.^102^ DPP9 may facilitate mitotic entry by affecting ubiquitination of the transcription factor forkhead box protein M1b (FoxM1b), a known substrate of PLK1.^103^ Future studies that explore the role of these proteins may also point toward new therapeutic avenues for the treatment of PLK1-overexpressing cancers. As such, our systematic integration of multiple unbiased platforms has led to several testable models for cancer-related discovery science, and we expect that the research community will benefit from our extensive validation strategies.

### Limitations of the study

This study provides compelling evidence for IGF2BP2 as a target in PLK1-overexpressing cancers. Although we validated key hits in patient-derived xenograft (PDX) models of breast and multiple breast cancer cell lines, the generalizability of these findings across all PLK1-driven cancers remains to be further tested in a broader panel of tumor types. Another limitation lies in the pharmacological targeting of IGF2BP2. While our small-molecule inhibitor (C4) demonstrated promising anti-tumor effects, its relatively high concentration in the micromolar range and moderate pharmacokinetic properties suggest that further optimization is needed to improve potency and bioavailability before clinical translation. Additionally, while C4 effectively reduced PLK1 levels and impaired mitochondrial function, its specificity for IGF2BP2 over other RNA-binding proteins should be further validated to rule out off-target effects.

## Resource availability

### Lead contact

Further information and requests for reagents and resources should be directed and will be fulfilled by the lead contact: Franco Vizeacoumar: franco.vizeacoumar@usask.ca.

### Materials availability

This study did not generate new unique reagents.

### Data and code availability

The data files were uploaded to the Gene Expression Omnibus (GEO) database and the SuperSeries accession number GEO: GSE203242. This reference consists of two subseries for raw microarray data (.CEL files) for the shRNA screen GEO: GSE203237, and deep sequencing data from the CRISPR screen (.fastq.gz files) GEO: GSE203240. All other data reported in this paper will be shared by the lead contact upon request. This study did not report the original code. Any additional information required to reanalyze the data reported in this paper is available from the lead contact upon request.

## Acknowledgments

We thank the members of the Vizeacoumar and Freywald laboratories for their insights and comments on the manuscript. We thank Dr. Alana L. Welm, University of Utah, for providing the HCI-010 PDX and related technical advice. We thank Dr. Laurent Creancier, Pierre-Fabre, for providing the HCT116-inducible PLK1 cells. We thank Janine Schreiber and Jennifer Wolf for their technical assistance. The study was approved by the Animal Ethics Research Board of the University of Saskatchewan, appropriate regulatory authorities (# 20150067), and the ethical board of the Niedersächsisches Landesamt für Verbraucherschutz und Lebensmittelsicherheit, Oldenburg, Germany.

Financial support was provided by 10.13039/501100000024Canadian Institutes of Health Research (CIHR) operating grants PJT-156309, PJT-156401, and PLL-192134 (F.J.V. and A.F.); 10.13039/100009326Cancer Research Society (CRS) operating funds 2017-OG-22493 (F.J.V. and A.F.); Be Like Bruce Foundation (F.J.V. and A.F.); 10.13039/501100001659Deutsche Forschungsgemeinschaft #453246190 (A.K.K. and M.E.); Canada Foundation for Innovation CFI-33364 (F.J.V.); Saskatchewan Cancer Agency operating grants from Cancer Foundation of Saskatchewan (F.J.V.); Lisa Rendall Fellowship (C.E.C.); College of Medicine, 10.13039/100008920University of Saskatchewan (UofS) (F.S.V.); Mitacs Globalink Fellowship (L.K.); SHRF Fellowship (R.D.); UofS CoMGRAD Award (V.M., H.P., and H.E.); 10.13039/501100000024CIHR – Canada Graduate Scholarships – Master’s (J.D.W.P.); UofS Health Science Graduate Scholarship (J.D.W.P.); CRS – Doctoral Research Award (J.D.W.P.); and CIHR – Canada Graduate Scholarships – Doctoral FBD-187665 (V.M.).

## Author contributions

Conceptualization, F.S.V., M.E., A.K.K., A.F., and F.J.V.; investigation, C.E.C., F.S.V., Y.Z., L.K., S.B., V.M., H.D., J.D.W.P., P.G., K.W., Y.W., M.L.-W., A.G., R.H., C.D., J.P.V., A.M.M., F.K., K.K.B., S.M., A.C., T.K., B.G.E.Z., K.R., P.W., L.G., H.P., H.E., R.D., O.A., A.D., T.F., E.P.M., E.R., J.S.L., K.R., M.K., L.H., C.H.L., S.Y., A.K., R.D., J.M., B.T., K.B., D.W.C., L.A., and Y.W.; writing – reviewing and editing, all authors; funding acquisition, A.F., A.K.K., M.E., and F.J.V.

## Declaration of interests

D.W.C reports consultancy and advisory relationships with AstraZeneca, Daiichi Sankyo, Exact Sciences, GenomeRx, Gilead, GlaxoSmithKline, Inivata/NeoGenomics, Lilly, Merck, Novartis, Pfizer, Roche, and SAGA and research funding to their institution from AstraZeneca, GenomeRx, Guardant Health, Grail, Gilead, GlaxoSmithKline, Inivata/NeoGenomics, Knight, Merck, Pfizer, ProteinQure, and Roche.

## STAR★Methods

### Key resources table

**Table:** 

REAGENT or RESOURCE	SOURCE	IDENTIFIER
**Antibodies**		

Rabbit GAPDH Antibody (FL-335)	Santa Cruz Biotechnology	sc-25778
Rabbit Plk Antibody (H-152)	Santa Cruz Biotechnology	sc-5585
Mouse Anti-CRISPR-Cas9 antibody [7A9-3A3]	Abcam	ab191468
Mouse Anti-IGF2BP2/IMP-2 antibody	Abcam	ab128175
Mouse Anti-Tubulin	Sigma-Aldrich	T9028
Rabbit Anti-PARP	Cell Signaling Technology	9542S

**Chemicals, peptides, and recombinant proteins**		

Dulbecco’s Modified Eagle Medium (DMEM)	Cytiva	SH30243.01
Dulbecco’s Phosphate Buffered Saline	Gibco	14190–144
Penicillin Streptomycin Solution, 100X	Corning	30-002-CI
RPMI-1640 Medium	Cytiva	SH30027.01
McCoy’s 5A Medium	Gibco	16600–082
Fetal Bovine Serum	Corning	35-077-CV
0.25% Trypsin, 2.21mM EDTA, 1X [-] sodium bicarbonate	Corning	25-053-CI
Opti-MEM™ I Reduced Serum Medium	Gibco	31985062
X-tremeGENE™ 9 DNA Transfection Reagent	Roche	06365809001
Albumin, Bovine Serium [BSA], Fraction V	Biomatik	A2134
Polybrene	Sigma-Aldrich	TR-1003
Blasticidin	InvivoGen	Ant-bl-1
Puromycin	InvivoGen	Ant-pr-1
2-hydroxypropyl-β-cyclodextrin	Sigma-Aldrich	332607
Doxycycline	Sigma-Aldrich	D9891
Formaldehyde	Fisher Scientific	F79
Crystal Violet	Sigma-Aldrich	61135
GAPDH probe	Thermo Scientific	VIC-MGB, Hs02786624_g1
IGF2BP2 probe	Thermo Scientific	FAM-MGB, Hs00538954_g1
PLK1 probe	Thermo Scientific	FAM-MGB, Hs00983227_m1
Resazurin	R&D systems	AR002
XhoI	New England Biolabs	R0146S
MammoCult™ Human Medium Kit	StemCell	05620

**Critical commercial assays**		

Mycoplasma PCR Detection Kit	Applied Biological Materials Inc.	G238
GeneArt™ Genomic Cleavage Detection Kit	Invitrogen	A24372
QIAamp Blood Maxi Kit	Qiagen	51194
High-Capacity cDNA Reverse Transcription Kit	Applied Biosystems	4368814
Chromium Next GEM Single-cell 3′ Reagent Kit	10X Genomics	PN-1000120
ddPCR™ Supermix for Probes	Bio-Rad	1863026

**Deposited data**		

All data SuperSeries	This study	GSE203242
CRISPR screens deep sequencing data (.fastq.gz files)	This study	GSE203240
shRNA screens raw microarray data (.CEL files)	This study	GSE203237

**Experimental models: Cell lines**		

BT549	Gift from Deborah Anderson, Saskatchewan Cancer Agency (SCA)	N/A
MDA-MB-231	ATCC	CRM-HTB-26
MCF7	Gift from Deborah Anderson, SCA	N/A
HCC1143	Gift from Deborah Anderson, SCA	N/A
Hs578Bst	ATCC	HTB-125
MCF10A	Gift from Deborah Anderson, SCA	N/A
HGF-1	ATCC	CRL-2014
HCT116-PLK1	Gift from Laurent Creancier, Pierre-Fabre, France	N/A
HCI-010 PDX	Gift from Alana Wehlm, Huntsman Cancer Institute PDX repository	N/A
HEK293T	Gift from Jason Moffat, University of Toronto	N/A

**Experimental models: Organisms/strains**		

Mouse: NOD.Cg-Prkdscidll2rg	Animal Care and Research Support, University of Saskatchewan	N/A
Mouse: CD-1 IGS	Charles River	022

**Oligonucleotides**		

Table S8	This study	N/A
Table S9	This study	N/A

**Recombinant DNA**		

psPAX2	Gift from Jason Moffat	N/A
pMD2.G	Gift from Jason Moffat	N/A

**Software and algorithms**		

Bayesian Analysis of Gene Essentiality	Hart, T. & Moffat, J., 2016	https://github.com/hart-lab/bagel
Gene Set Enrichment Analysis	UC San Diego, Broad Institute	https://www.gsea-msigdb.org/gsea/index.jsp
FastQC version 0.11.9	Babraham Bioinformatics	https://www.bioinformatics.babraham.ac.uk/projects/fastqc/
Bowtie2 version 2.5.0	Langmead and Salzberg, 2012	http://bowtie-bio.sourceforge.net/bowtie2/index.shtml
Cell Ranger 6.1.2	10X Genomics	https://www.10xgenomics.com/support/software/cloud-analysis/latest/release-notes/CA-release-notes-archive
Loupe Browser 5.0	10X Genomics	https://www.10xgenomics.com/support/software/loupe-browser/latest
ImageJ	NIH, USA	https://imagej.nih.gov/ij/
QuantaSoft software	Bio-Rad	https://www.bio-rad.com/en-ca/life-science/digital-pcr/qx200-droplet-digital-pcr-system/quantasoft-software-regulatory-edition
MultiQuant software	Sciex	https://sciex.com/products/software/multiquant-software
Prism9	GraphPad Inc.	https://www.graphpad.com/scientific-software/prism/

**Other**		

EVOS fluorescence microscope	Invitrogen	
QX100 ddPCR Droplet generator	Bio-Rad	
CFX96 Real-Time PCR Thermocycler	Bio-Rad	
Varioskan LUX Plate Reader	Thermo Fisher	
IncuCyte S3 Live Cell Imager	Sartorius	
Image Xpress	Molecular Devices	
Biomek FX	Beckman Coulter	
NovaSeq 6000 S1 Flow Cell	Illumina	Global Institute for Food Security
Chromium Next GEM Instrument	10X Genomics	Global Institute for Food Security

### Experimental model and study participant details

#### Cell lines and cell culture

All the cell lines used in this study are listed in Table S10. Cells were incubated at 37°C and 5% CO2. All cell lines were purchased from Cedarlane Labs, unless otherwise indicated (Burlington, Ontario, Canada), a Canadian distributor for American Type Culture Collection (ATCC), or Millipore Sigma. Cell lines purchased from ATCC/Sigma were passaged for less than three months at a time following resuscitation; therefore, no additional authentication was performed. Mycoplasma tests were routinely performed.

#### Mouse models, tumor xenograft and pharmacokinetic studies

All animals in xenograft experiments were handled in accordance with protocols approved by the University of Saskatchewan Animal Research Ethics Board (AREB). The mice used in xenograft experiments were obtained from an established colony of immunodeficient NOD.Cg-*Prkdc*^*scid*^ *Il2rg*^*tm1Wjl*^/SzJ (NSG) mice. NSG mice were obtained from the Laboratory Animal Services Unit (LASU) at the University of Saskatchewan and randomly allocated to experimental groups. The indicated cells were trypsinized and resuspended in ice-cold PBS. MDA-MB-231 cells were injected into the inguinal mammary fat pads of 6–10-week-old female NSG mice (2x10^6^ cells per mouse). DMSO and C4 were dissolved in PBS containing 5% 2-hydroxypropyl-β-cyclodextrin (HP-β-CyD) as an excipient and injected intraperitoneally five times per week for four weeks (DMSO, *n* = 10; C4, *n* = 8) in a total volume of 100 μL.

Treatment with an IGF2BP2 inhibitor (C4) was initiated on the day after the injection of MDA-MB-231 cells. Injections were given for five consecutive days, followed by two days of rest, for a total of 30 days. Tumors were measured every three–four days using a digital caliper, and the tumor volume was calculated using the formula (A × B^2^)/2, where A and B represent the long and short diameters of the tumor, respectively.

For the pharmacokinetic studies, 4-week-old outbred male CD-1 mice (Charles River, Germany) were used. Animal studies were conducted at the Helmholtz Center for Infection Research (Department of Chemical Biology) in accordance with the recommendations of the European Community. All animal procedures were performed in strict accordance with the German regulations of the Society for Laboratory Animal Science (GV-SOLAS) and the European Health Law of the Federation of Laboratory Animal Science Associations (FELASA). Animals were excluded from further analysis if sacrifice was necessary, according to the human endpoints established by the ethical board. All pharmacokinetic experiments were approved by the ethical board of the Niedersächsisches Landesamt für Verbraucherschutz und Lebensmittelsicherheit, Oldenburg, Germany. Compounds C1, C4, C6, and C9 were dissolved in 1.6% DMSO, 20% PEG400, 20% Tris (1%, pH 9.0), and 58.4% 0.9% isotonic NaCl solution. Mice were administered C1, C4, C6, and C9 in cassette PK format at 1 mg/kg IV per compound. Approximately 20 μL of whole blood was collected serially from the lateral tail vein at 0.25, 0.5, 1, 2, 4, and 8 h post-administration. After 24 h, the mice were euthanized, and blood was collected from the heart. Whole blood was collected into Eppendorf tubes coated with 0.5 M EDTA. One part was immediately spun at 13.000 rpm for 10 min at 4°C. The plasma was transferred into a new Eppendorf tube and stored at −80°C until analysis. Additionally, the blood cells and whole blood were preserved and used for analysis. Furthermore, C4 and C6 were tested in a single-dose PK study with 10 mg/kg IP. C4 and C6 were dissolved in 10% DMSO and 90% Tris (1%, pH 9.0), respectively. Approximately 20 μL of whole blood was collected serially from the lateral tail vein at 0.25, 0.5, 1, 6, 24, and 48 h post-administration for C4 and at 0.25, 0.5, 1, 2, 4, and 8 h post-administration for C6. After 72 h for C4 mice and after 24 h for C6 mice, blood was collected from the heart, and whole blood was collected into Eppendorf tubes coated with 0.5 M EDTA and immediately centrifuged at 13,000 rpm for 10 min at 4°C. The plasma was transferred into a new Eppendorf tube and stored at −80°C until analysis. Spontaneous urine was collected from the cassette and from single-dose studies. Liver samples were homogenized in an isotonic sodium chloride solution using a Polytron homogenizer.

All PK plasma samples were analyzed by HPLC‒MS/MS using an Agilent 1290 Infinity II HPLC system coupled to an AB Sciex QTrap6500plus mass spectrometer. First, a calibration curve was prepared by spiking different concentrations of C1, C4, C6, and C9 into mouse plasma, whole blood, blood cells, and urine from CD-1 mice. Caffeine was used as the internal standard. Quality control samples (QCs) were prepared for C1, C4, C6, and C9 in their respective matrices. The same extraction procedure was used: 7.5 μL of a plasma or whole-blood sample, 5 μL of a blood cell sample +7.5 μL of isotonic sodium chloride solution, or 10 μL of a urine sample (from the calibration samples, QCs, or PK samples) was extracted with 37.5 μL of methanol containing 12.5 ng/mL caffeine as an internal standard for 10 min at 2000 rpm on an Eppendorf MixMate vortex mixer. The samples were then spun at 13,000 rpm for 10 min at 4°C. The supernatants were then transferred to standard HPLC glass vials. 50 μL of a homogenized liver sample (adjusted to a final concentration of 300 mg/mL; calibration samples, QCs or PK samples) was extracted with 50 μL of methanol containing 12.5 ng/mL caffeine as an internal standard for 10 min at 800 rpm on an Eppendorf MixMate vortex mixer. The samples were then spun at 4,000 rpm for 10 min at 4°C. Supernatants were transferred to 96-well V-bottom Greiner plates and sealed. The HPLC conditions used were as follows: column: Agilent Zorbax Eclipse Plus C18, 50 × 2.1 mm, 1.8 μm; temperature: 30°C; injection volume: 1 μL; flow rate: 700 μL/min; solvent A: water +0.1% formic acid; solvent B: acetonitrile +0.1% formic acid; gradient: 99% A at 0 min and until 0.1 min 99% - 0% A from 0.1 min to 4.0 min, 0% An until 4.5 min, 0%–99% A from 4.5 min to 4.7 min. The mass spectrometric conditions were as follows: scan type: MRM, positive and negative mode; Q1 and Q3 masses for caffeine, C1, C4, C6, and C9 are shown in Table S11. The peak areas of each sample and the corresponding internal standard were analyzed using MultiQuant 3.0 software (AB Sciex). PK parameters were determined by non-compartmental analysis using PKSolver.^104^

### Method details

#### Transfections and transductions

Lentiviral particles were generated by transfecting HEK293T cells with psPAX2 and pMD2.G, and a pLKO.1-shRNA 9:1:10 plasmid mixture was generated using Xtremegene 9 transfection reagent (Roche) and Opti-MEM media (Gibco). The medium was replaced with DMEM containing 2% (w/v) bovine serum albumin 18 h post-transfection, and the lentivirus-containing media were harvested at 48- and 72-h post-transfection. Transfection with siRNA for Hs578Bst, Hs895-Sk, HGF1, MCF10A and MDA-MB-231 was performed using RNAiMax transfection reagent (Life Technologies) and Opti-MEM to a final siRNA concentration of 50 nM Cas9-expressing HCI-010 cells were generated by transducing HCI-010 cells with Cas9-blast lentiviral particles (Addgene #52962), replacing the lentiviral medium after 24 h, and adding 5 μg/mL blasticidin 48 h after transduction for 21 days. Antibodies against GAPDH (sc-25778) and PLK1 (sc-5585) were obtained from Santa Cruz, Cas9 (ab191468) and IGF2BP2 (ab128175) from Abcam, tubulin (#T902) from Merck, and PARP (9542S) from Cell Signaling.

#### Genome-wide pooled shRNA screening and data analysis

Screening and microarray scoring were performed as previously described.^48^ HCT116-PLK1 cells were transduced at an MOI of 0.3, and 24 h after transduction, the cells were treated with 2 μg/mL puromycin for 48 h. The puromycin-selected cells were divided into two populations, and one population was induced with doxycycline (PLK1-IN) on day 0. The cells were passaged over 16 days with 200x hairpin representation and samples were collected on days 0, 8, and 16. Genomic DNA was extracted at each time point, and shRNA sequences were PCR-amplified at 98°C for 3 min, followed by 29 cycles of 98°C for 10 s, 55°C for 15 s, and 72°C for 15 s. Amplified hairpins were digested with XhoI and the stable half-hairpin gel was extracted, purified, and subjected to probe hybridization on UT-GMAP 1.0. The signal intensities for the microarray were normalized via quantile normalization, and shRNAs with signals below the background (i.e., a log_2_ scale of less than 8) at the initial timepoint T0 and signals below 0 at T8 and T16 were removed prior to further analyses to compute the fitness score. The weighted differential cumulative change (WDC_h_) for each shRNA between doxycycline-induced cells (PLK1-IN) and its isogenic uninduced cells (PLK1-UN) was calculated for each consecutive time point using the following formula:

$${WDC}_{h}=\sum_{t=0}^{T}\frac{\varepsilon }{t+1}\left(x_{t+1,r}^{PLK1-IN}-x_{t,r}^{PLK1-IN}\right)-\sum_{t=0}^{T}\frac{\varepsilon }{t+1}\left(x_{t+1,r}^{PLK1-UN}-x_{t,r}^{PLK1-UN}\right)$$

where $x_{t,r}^{PLK1-IN}$ is the normalized signal intensity of the PLK1-IN cells at time point t∈(0,..T) in replicates of r∈(1..N). Similarly, $x_{t,r}^{PLK1-UN}$ represents PLK1-UN cells. ε is a constant that determines the weight between each time point so that shRNA drops at earlier time points are ranked before shRNA drops at later time points. The WDC_gene_ gene was computed as the average of the two shRNAs with the most negative values for that gene using the following formula:

$${WDC}_{gene}=average\left(\arg_{h,h^{\prime }}^{\min}\left[{WDC}_{gene,h}\;;{WDC}_{gene,h^{\prime }}\right]\right)$$

To identify shRNAs and their corresponding genes that are significantly different between PLK-IN and PLK1-UN cells, Student’s t test was used in combination with the permutation test *p* value by estimating the frequency of randomized, shuffled WDCs with more negative values in comparison with the observed gene-level WDC value, as previously described.^48^ Bayesian analysis of the gene essentiality algorithm was used to evaluate the performance of the screens.^105^

#### Computational analysis and datasets

Reactome pathway enrichment was performed by using the Reactome database to assign genes to pathways (https://reactome.org/),^106^ after which gene enrichment analysis was performed on the query gene set of 960 PLK1 screening hits using the idep software package.^107^ Gene expression analysis was performed using The Cancer Genome Atlas database downloaded from the Genomic Data Commons data portal (https://portal.gdc.cancer.gov/) for 33 different cancer types. The RNA-Seq Expectation Maximization (RSEM)-normalized mRNA expression data were log2 transformed, and the Spearman-rank correlation between PLK1 and each of the 960 SDL hits was calculated for each of the 33 different malignant patient samples. The resulting correlation coefficient (r) was subsequently clustered by calculating the Euclidean distance between the different genes.

The gene expression profile from the Cancer Cell Line Encyclopedia database^64^ was used to rank cell lines according to PLK1 expression and classify them into two groups of cell lines that overexpressed or underexpressed PLK1. For the essentiality datasets from Project Achilles^65^ and Marcotte et al.*,*^62^ the top 25% and bottom 25% of the ranked cell lines were assigned as PLK1-overexpressing and under-expressing, respectively. To determine whether a PLK1 screening hit was classified as essential or non-essential, the fold change in the essentiality scores between the PLK1-overexpressing and PLK1-underexpressing groups, as well as the *p*-value, was calculated using the Mann-Whitney U test.

ToppGene candidate gene prioritization^69^ was performed using “PLK1” as the training gene set and the list of 960 PLK1 screening hits as the test gene set. The ToppGene database was used to compile functional annotations and network analysis results from Gene Ontology (GO) for molecular function, biological process, cellular component, gene expression, pathway, protein domain, transcription factor-binding site, miRNA target, drug–disease interaction, disease–drug interaction, and interaction information published in the NCBI search engine PubMed. Gene expression data from TCGA were used to divide the patients into two groups based on PLK1 expression and PLK1 screening hit expression for each of the 960 PLK1 screening hits. The median gene expression values for PLK1 and for the screening hits were determined and used as cutoff values to divide the samples into PLK1 low and PLK1 high. TCGA clinical data were subsequently subjected to standard Kaplan-Meier analysis between patients who exhibited natural SDL and those who did not.

Drug response analysis was performed using the Genomics of Drug Sensitivity in Cancer (GDSC) dataset. GDSCs are involved in gene expression and responses to various drugs in several cancer cell lines. We grouped the cell lines based on the expression of PLK1 in the top 1% and bottom 1%. We then computed the *p*-value for the difference in the *p*-value between the PLK1-high and PLK1-low cells using the Mann‒Whitney U test for the IC50s of the inhibitors of the SDL hit present in the GDSC.

#### Pooled *in vivo* CRISPR screening

The mice were housed under sterile conditions at the University of Saskatchewan, and all animal protocols were reviewed and approved by the University of Saskatchewan Animal Research Ethics Board. Digital caliper measurements were used to monitor tumor size, and tumor volume was calculated using the formula A/2 × B2 (where A and B are the long and short diameters, respectively). The sgRNA sequences used in the imaging experiment were pooled and used to generate a lentiviral library. HCI-010 and HCI-010+Cas9 PDX-derived cells^70^ were transduced at an MOI of 0.3, and after 24 h, they were selected using 2 μg/mL puromycin. After 48 h of selection, 3 million viable cells mixed 1:1 with Matrigel (Corning) in a total volume of 100 μL were injected into the mammary fat pads of 6–8-week-old female mice and allowed to grow for 3 weeks. Tumors were harvested and flash-frozen in liquid nitrogen. Genomic DNA was extracted using a mortar and pestle and DNA Blood Maxi Kit (Qiagen, Hilden, Germany). A sequencing library was constructed as described previously^108^ with slight modifications to the primers of the first PCR to match the vector backbone^109^ (forward primer 5-caaaatacgtgac gtagaaagtaataatttcttgggtag-3′ and reverse primer 5′-gcgtaaaattgacgcatgt gttttatcggtctgtatatcgag-3′). Fastq files were aligned to the sequence library using the Bowtie2 alignment package. Alignment with scores above the default threshold score was mapped to the library. The count matrix data were analyzed as described in the above pooled screen analyses.

#### Microscopy and imaging

HCI-010 and HCI-010+Cas9 cells were seeded in 96-well low-attachment plates at a concentration of 10 000 cells per well with 8 μg/mL polybrene and 100 μL of sgRNA-lentiviral targeted pool (two sgRNAs for each target gene and one gene per well) in a total volume of 200 μL. After 24 h, the medium was replaced with a medium containing 2 μg/mL puromycin. After 48 h of puromycin selection, four sites per well were imaged with a 4× objective using the brightfield setting for several days (day 0, day 4, and day 6). After imaging, the images were analyzed using the MetaXpress custom module, in which the cells were carefully identified by eliminating any artifacts. A custom algorithm was also used to measure the area, perimeter, and number of cells per well. The increase in cell confluency each day was compared to baseline (day 2), and HCI-010+Cas9 was subsequently compared to HCI-010 to determine whether the gene knockouts caused a decrease in cell proliferation.

#### Direct capture of guide RNA using a perturb-seq screen

The 10x Genomics Chromium Next GEM Single-cell 3′ Reagent Kit-compatible sgRNA library, which included 130 sgRNAs (targeting 65 genes), 15 non-targeting controls, and 16 positive controls, was generated by MilliporeSigma. Samples for direct-capture single-cell perturb-seq were prepared by transducing MCF7-Cas9 cells with single-cell perturb-seq sgRNA library lentiviral particles and harvested 2 days after puromycin selection. The 3′Gene Expression Library and CRISPR Screening Library were constructed following the instructions of the Chromium Next GEM Single-cell 3′Reagent Kit v3.1 (10x Genomics). Both libraries were sequenced on the NovaSeq S1 Flow Cell platform (Illumina). Raw data from the NovaSeq platform were extracted as Fastq.gz files. These files were mapped to the GRC38 reference genome and aligned using the cell ranger (v6.1.2) count algorithm, run on 32 core cluster computing datasets. The samples were split into three batches with a cell expectancy of approximately 10,000 cells per batch. The three batches were pooled using the cell ranger aggregation algorithm. Further investigation revealed that several cells had more than one sgRNA. Hence, cells with a single sgRNA were curated and recomputed using the Cell Ranger reanalysis pipeline. The resulting output was then imported into Loupe Browser (v.6.0) to generate various visualization outputs.

#### Colony formation

The cells (1x10^3^/well for MCF7 cells, MDA-MB-231 cells, and 5x10^3^/well for BT-549 cells) were seeded in 6-well plates and incubated at 37°C under 5% CO_2_ for 10 days, during which the medium was replaced every 3 days. The plates were washed with PBS, fixed with 4% formaldehyde, and stained with 0.5% crystal violet. Colonies were counted using ImageJ software and images of the colonies were scanned.

#### Genomic cleavage detection

Primers for genomic cleavage detection were designed for the region around the sgRNA target sequence with the forward primer ∼200 bp upstream and the reverse primer ∼300 bp downstream using Primer3 v. 0.4.0 (http://bioinfo.ut.ee/primer3-0.4.0/). The primer sequences are provided in Table S12. The cells were lysed, the sgRNA target region was PCR-amplified, and mismatched enzymes were digested with T7 endonuclease using a GeneArt Genomic Cleavage Detection Kit according to the manufacturer’s instructions (Invitrogen).

#### Droplet digital PCR

cDNA templates for droplet digital PCR were prepared using the High-Capacity cDNA Reverse Transcription Kit (Applied Biosystems). The 20 μL quantitative polymerase chain reaction was performed using 10 μL ddPCR Supermix for Probes (Bio-Rad), 1 μL of target gene probe (*IGF2BP2*: FAM-MGB, Hs00538954_g1, *PLK1*: FAM-MGB, Hs00983227_m1, Thermo Scientific), 1 μL of reference probe (*GAPDH*: VIC-MGB, Hs02786624_g1, Thermo Scientific), and 50 ng of cDNA template. Droplets were generated using a QX100 ddPCR droplet generator (Bio-Rad Laboratories). Quantitative PCR was performed on a CFX96 Real-Time PCR Thermocycler (Bio-Rad Laboratories) using the following program: 95°C for 10 min, 45 cycles of 94°C for 30 s, 60°C for 90 s, 98°C for 10 min after 45 cycles, and 4°C on hold. The signals were detected using a QX100 ddPCR Droplet Reader (Bio-Rad Laboratories), and absolute quantification was performed using QuantaSoft software (Bio-Rad Laboratories).

#### Mitochondrial stress test

Mitochondrial stress test assays were performed using a Seahorse 96XF analyzer (Agilent). The Seahorse XF Cell Mito Stress Test Kit was used according to the manufacturer’s protocol. 30,000 cells/well were seeded into a Seahorse XF Cell Culture Microplate one day before the assay and incubated overnight. Four hours before the assay, cells were treated with 50 μM of IGF2BP2 inhibitors or a respective solvent control. During the assay cells were treated with 1.5 μM oligomycin, 0.5 μM carbonyl cyanide-*p*-trifluoromethoxy-phenylhydazone (FCCP), and 0.5 μM rotenone/antimycin A. After assay completion, cells were stained with 5 μg/mL of Hoechst dye to detect the cell number by measuring the fluorescence intensity in a Cytation 5 plate reader (BioTek). Data were analyzed using Seahorse Wave software (Agilent) to calculate oxygen consumption rate (OCR) and extracellular acidification rate (ECAR).

#### RNA sequencing and data analysis

RNA of four-day-old spheroids (3D) or 80% confluent adherent (2D) HCT116 and HCT116 IGF2BP2 knockout cells^90^ was isolated using the High Pure RNA-Isolation Kit (Roche). RNA integrity was checked using the RNA 6000 Nano Kit (Agilent). RNAseq libraries were prepared using 100 ng of total RNA per sample with the NEBnext Ultra II Directional RNA library kit for Illumina (New England Biolabs) according to the manufacturer’s recommendations. Libraries were quantified with the NEBnext library quant kit for Illumina (NEB) and then sequenced for 100 nt using a V3 single read flow cell on a HiSeq 2500 (Illumina). After QC with FastQC version 0.11.2, reads were indexed with SAMtools version 1.3.1^110^ and adaptor-trimmed (Q < 20) with Cutadapt version 1.4.132 using Trim Galore! version 0.3.3. Reads were aligned to hg38 assembly with the grape-nf pipeline (https://github.com/guigolab/grape-nf) wrapping STAR version 2.4.0j33^111^ and RSEM version 1.2.2134.^112^ Differential analyses were performed in R using the DESeq2 package.^113^ Further analysis was carried out in the Ingenuity Pathway Analysis software IPA (Qiagen), ShinyGO 0.77^114^ & STRING version 12.0.^115^

#### Cell viability assay

The viability-inhibiting activities of the chemical compounds were assessed using a resazurin reduction assay. MDA-MB-231, MCF7, and BT-549 cells were treated with increasing concentrations of the drugs (from 5 to 75 μM) and incubated for 72 h before resazurin (R&D Systems) was added. Fluorescence was detected using a Varioskan LUX Plate Reader (Thermo Fisher Scientific). Each experiment was conducted thrice, each with six replicates. Dose–response curves were generated, and 50% inhibitory concentrations (IC_50_) were calculated using GraphPad Prism 9.

#### Proliferation assay in tumorspheres

The cells were seeded into 24-well ultra-low attachment plates (4x10^3^/well) in a complete MammoCult medium (StemCell) and allowed to propagate in tumorspheres for seven days. For each replicate of the IGF2BP2-KD cell line and the corresponding control (shRFP), tumorspheres from four independent wells were combined and dissociated using mechanical dissociation (10 replicates of each sample were pipetted on P1000), and proliferation was assessed by cell counting using a hemocytometer. Each sample was assessed in triplicate and three independent experiments were performed. Images were captured using the EVOS fluorescence microscope (Invitrogen). Images were converted to 8-bit binary images and analyzed using the ImageJ software.

#### Analyses of PDX models

The tumor growth patterns of 174 PDX models from various tissue types, such as breast, colorectal, non-small cell lung carcinoma, melanoma, and pancreatic tissue types that had matching RNA-Seq data, were taken from previously published studies.^88^ The samples of the 174 PDX models that were not exposed to any drug treatment were chosen for further analysis. The slope of the tumor growth curve was calculated using the Xeva package (v.1.99.20).^89^ The doubling time of the tumor was obtained from an original publication.^88^ The grouping of PDX models was based on RNA-Seq expression in fragments per kilobase of transcript per million mapped fragments (FPKM) units. An FPKM value < 20 was considered to indicate low expression and a value > 30 was considered to indicate high expression of both PLK1 and IGF2BP2, as most PDX samples showed high expression of PLK1. Accordingly, the PDX models were grouped into two categories: PLK1 high expression and IGF2BP2 low expression (representing SDL) and high expression of PLK1 and IGF2BP2. Finally, the slope and doubling time of each group were plotted in violin plots and their significance was calculated using an unpaired t-test with Welch’s correction.

#### Statistical analysis

GraphPad Prism software was used for all tests. Appropriate statistical method used in each case are explained in the corresponding methods section/figure legend. The significance of our results was determined by setting *p* < 0.05 and the error was reported as mean ± standard deviation (SD) when not specifically indicated. Patient survival analyses were performed using the Kaplan–Meier estimator with the non-parametric log rank test to measure the equity of strata. For xenograft work, an unpaired heteroscedastic t-test was used to assess statistical significance.

## Supplemental information

Document S1. Figures S1–S9

Table S1. SDL hits from the genome-wide, loss-of-function screen, related to Figure 1 List of SDL hits identified from the genome-wide shRNA screen with significant weighted differential cumulative changes in PLK-induced vs. PLK1-uninduced cells.

Table S2. Analyses of SDL hits from the genome-wide, loss-of-function screen, related to Figure 1 Comparison of all 960 hits with previously published screens associated with mitosis and cell cycle progression.

Table S3. DepMap-based prioritization of SDL hits from the genome-wide, loss-of-function data, related to Figure 1 List of genes with validated scores based on the work of Marcotte et al. or Project Achilles screens used to prioritize PLK1-SDL interactions. Significant p values indicate that PLK1 interacts with the SDL.

Table S4. Patient survival-based prioritization of SDL hits from the genome-wide, loss-of-function data, related to Figure 1 List of genes prioritized using clinical data to capture "naturally occurring" SDL interactions in patients across multiple cancer types.

Table S5. Prioritization of SDL hits from the genome-wide, loss-of-function data, related to Figure 1 List of genes prioritized from (1) ToppGene prioritization, (2) localized to the plasma membrane, and/or (3) located on the cytoband 19p13.2–3.

Table S6. Results from *in vitro* CRISPR screening, related to Figure 1 List of SDL hits validated by an *in vitro* CRISPR screening approach.

Table S7. Results from *in vivo* CRISPR screening, related to Figure 1 List of SDL hits validated by an *in vivo* CRISPR screening approach.

Table S8. Sequences of the guide RNAs used in the study, related to Figure 2 List of sgRNAs used as positive and negative controls in Perturb-seq screening.

Table S9. Differentially expressed gene list from RNA sequencing, related to Figure 3 RNA-sequencing analyses from 2D and 3D cultures.

Table S10. List of cell lines used in the study, related to STAR methods

Table S11. Output from mass spectrometric experiments, related to STAR methods

Table S12. List of primer sequences used for genomic cleavage detection, related to STAR methods

Document S2. Article plus supplemental information
