# Supplementary material for: Identification of targetable vulnerabilities of PLK1-overexpressing cancers by synthetic dosage lethality
Source: Cell Genom. 2025 May 9;5(6):100876. doi: 10.1016/j.xgen.2025.100876 (PMC12230241; doi:10.1016/j.xgen.2025.100876)
Supplement: Document S1. Figures S1–S9 [file mmc1.pdf]

Cell Genomics, Volume *5*

Supplemental information

Identification of targetable vulnerabilities of PLK1-overexpressing cancers by synthetic dosage lethality

Chelsea E. Cunningham, Frederick S. Vizeacoumar, Yue Zhang, Liliia Kyrylenko, Simon Both, Vincent Maranda, He Dong, Jared D.W. Price, Peng Gao, Konrad Wagner, Yingwen Wu, Mary Lazell-Wright, Ashtalakshmi Ganapathysamy, Rithik Hari, Kalpana K. Bhanumathy, Connor Denomy, Anjali Saxena, Jeff P. Vizeacoumar, Alain Morejon Morales, Faizaan Khan, Shayla Mosley, Angie Chen, Tetiana Katrii, Ben G.E. Zoller, Karthic Rajamanickam, Prachi Walke, Lihui Gong, Hardikkumar Patel, Hussain Elhasasna, Renuka Dahiya, Omar Abuhussein, Anton Dmitriev, Tanya Freywald, Erika Prando Munhoz, Eytan Ruppin, Joo Sang Lee, Katharina Rox, Martin Koebel, Laura Hopkins, Cheng Han Lee, Sunil Yadav, Gilles Gasparoni, Jörn Walter, Anand Krishnan, Raju Datla, Behzad Toosi, Kristi Baker, Jalna Meens, David W. Cescon, Laurie Ailles, Scot C. Leary, Yuliang Wu, Martin Empting, Alexandra K. Kiemer, Andrew Freywald, and Franco J. Vizeacoumar

Fig S1

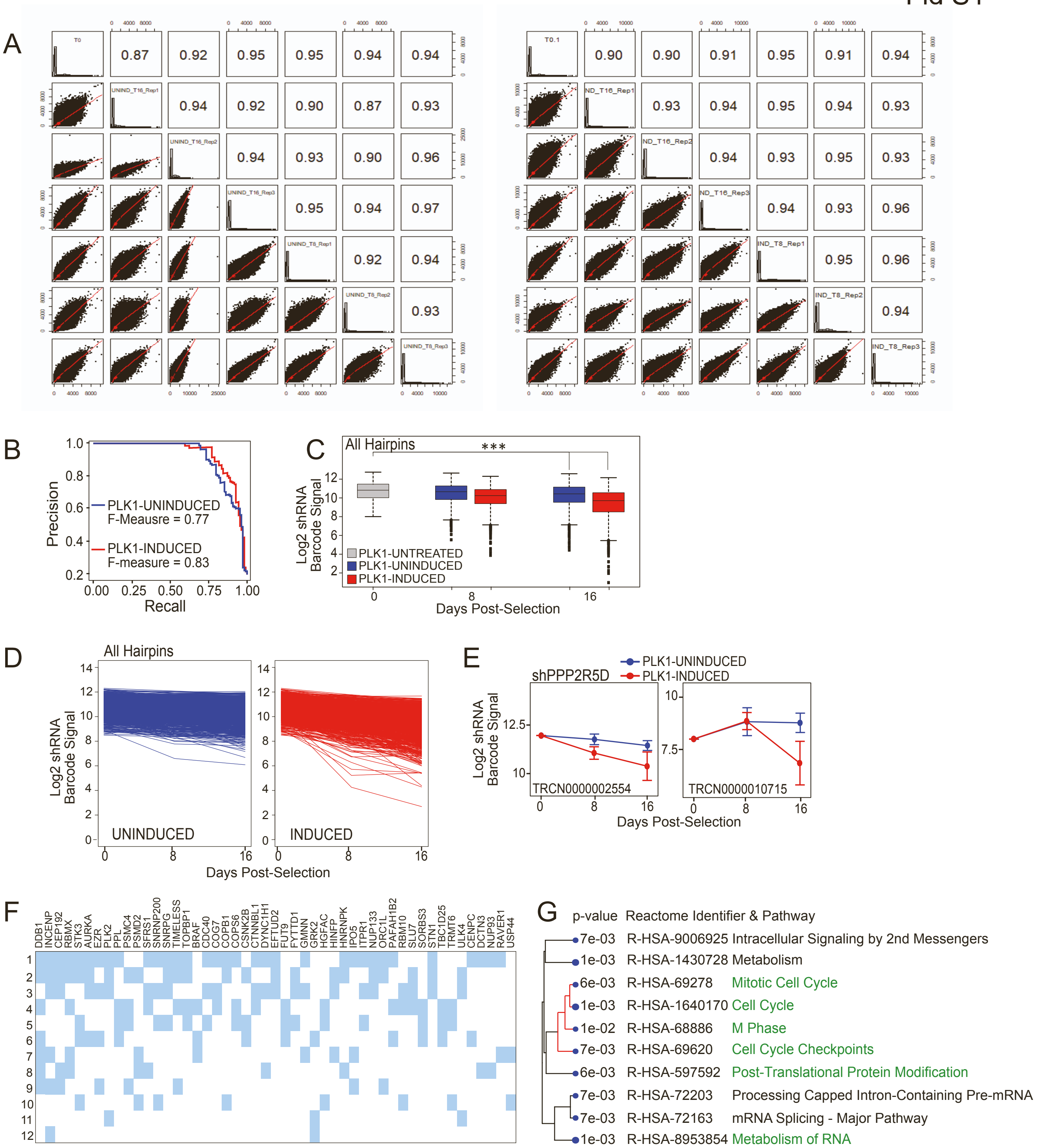

A
B
C
D
E
F
G
All Hairpins
PLK1-UNINDUCED
F-Meausre = 0.77
PLK1-INDUCED
F-measure = 0.83
Precision
Recall
Log2 shRNA Barcode Signal
PLK1-UNTREATED
PLK1-UNINDUCED
PLK1-INDUCED
Days Post-Selection
UNINDUCED
INDUCED
shPPP2R5D
TRCN0000002554
TRCN0000010715
p-value Reactome Identifier & Pathway
7e-03 R-HSA-9006925 Intracellular Signaling by 2nd Messengers
1e-03 R-HSA-1430728 Metabolism
6e-03 R-HSA-69278 Mitotic Cell Cycle
1e-03 R-HSA-1640170 Cell Cycle
1e-02 R-HSA-68886 M Phase
7e-03 R-HSA-69620 Cell Cycle Checkpoints
6e-03 R-HSA-597592 Post-Translational Protein Modification
7e-03 R-HSA-72203 Processing Capped Intron-Containing Pre-mRNA
7e-03 R-HSA-72163 mRNA Splicing - Major Pathway
1e-03 R-HSA-8953854 Metabolism of RNA

Figure S1. Screening quality analyses of the genome-wide, loss-of-function data, related to Figure 1. A. Correlations between replicates of uninduced and induced samples according to the genome-wide shRNA screens. Correlations between multiple timepoints are presented. The left panel shows the correlation between uninduced samples, and the right panel shows the correlation between induced samples. **B.** Precision vs. recall (PR) curve calculated by measuring the Bayes factor for the genes previously described as general essential and non-essential genes from published screens. F-measure > 0.75. **C.** Box plots summarizing microarray signals for all queried hairpin barcodes at different timepoints in PLK1-untreated (no induction) and PLK1-induced conditions. A 2.2-fold decrease in the induced population from T0 to T16 was observed (Kolmogorov–Smirnov test $p<0.0001$). **D.** Magnitudes of dropouts between uninduced and induced samples at different time points for each hairpin from the genome-wide shRNA screen are plotted. **E.** Drop plots of the microarray signals for the two individual hairpins targeting the PPP2R5D gene over time in the PLK1-uninduced and PLK1-induced strains. **F.** SDL hits that were also picked up from published mitosis-related screens. Only the 45 genes with the most overlap are shown. Rows and columns are sorted by the total number of overlaps in descending order. The references for each row are as follows: 1:PMID: 20360068; 2:PMID: 15616564; 3:PMID: 20360735; 4, 5, 6:PMID: 24104479 in MUS81; BLM; and PTTG1 null cells; 7:PMID: 27929715 in U2OS cells; 8:PMID: 14654843; 9:PMID: 27929715 in RPE1-hTERT cells; 10:PMID: 24104479 in PTEN null cells; 11:PMID: 16564017; and 12:PMID: 17001007. **G**. Dendrograms of the Reactome pathways that are significantly enriched for the 960 PLK1-SDL candidate genes. FDR-adjusted p values are indicated in the figure.

Fig S2

A

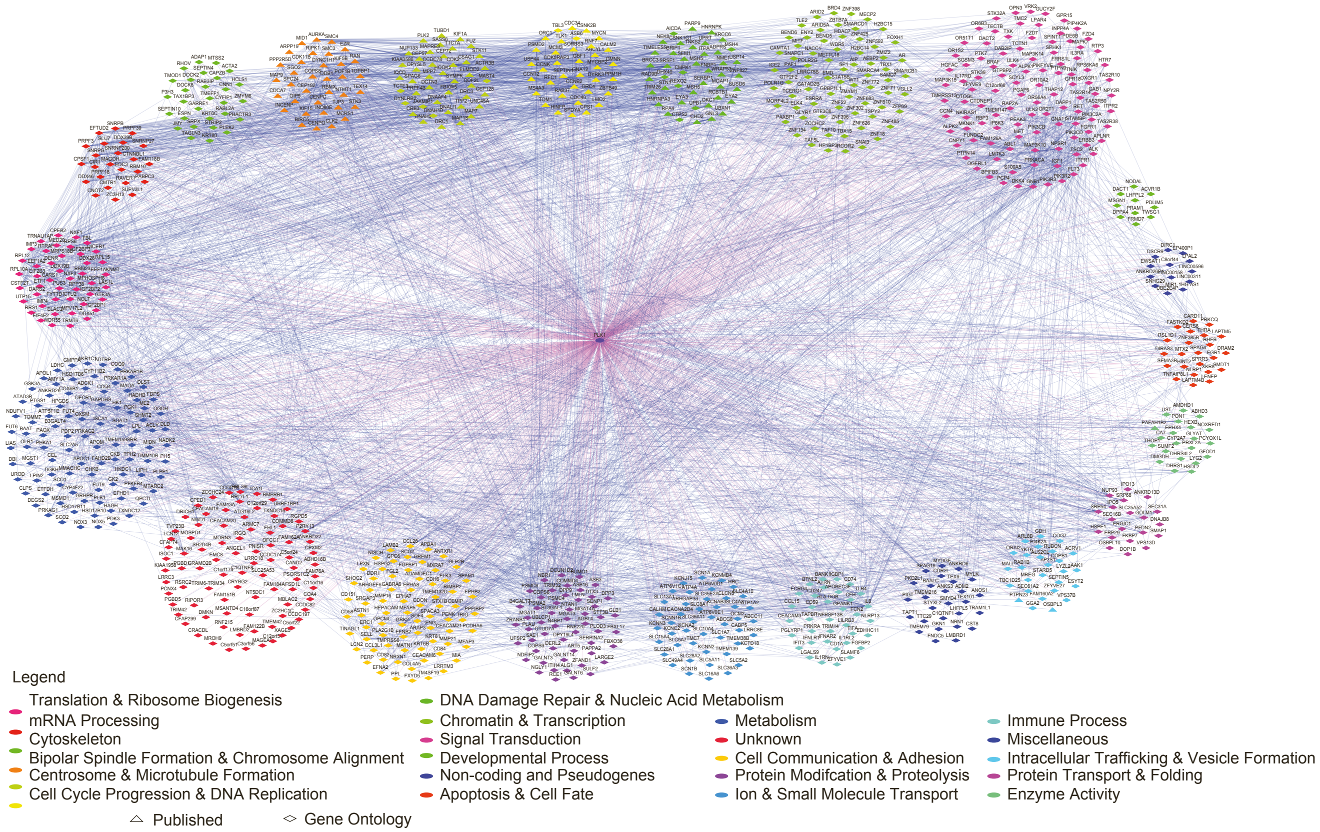
PLK1
Legend
Translation & Ribosome Biogenesis
mRNA Processing
Cytoskeleton
Bipolar Spindle Formation & Chromosome Alignment
Centrosome & Microtubule Formation
Cell Cycle Progression & DNA Replication
DNA Damage Repair & Nucleic Acid Metabolism
Chromatin & Transcription
Signal Transduction
Developmental Process
Non-coding and Pseudogenes
Apoptosis & Cell Fate
Metabolism
Unknown
Cell Communication & Adhesion
Protein Modifcation & Proteolysis
Ion & Small Molecule Transport
Immune Process
Miscellaneous
Intracellular Trafficking & Vesicle Formation
Protein Transport & Folding
Enzyme Activity
Published
Gene Ontology

B

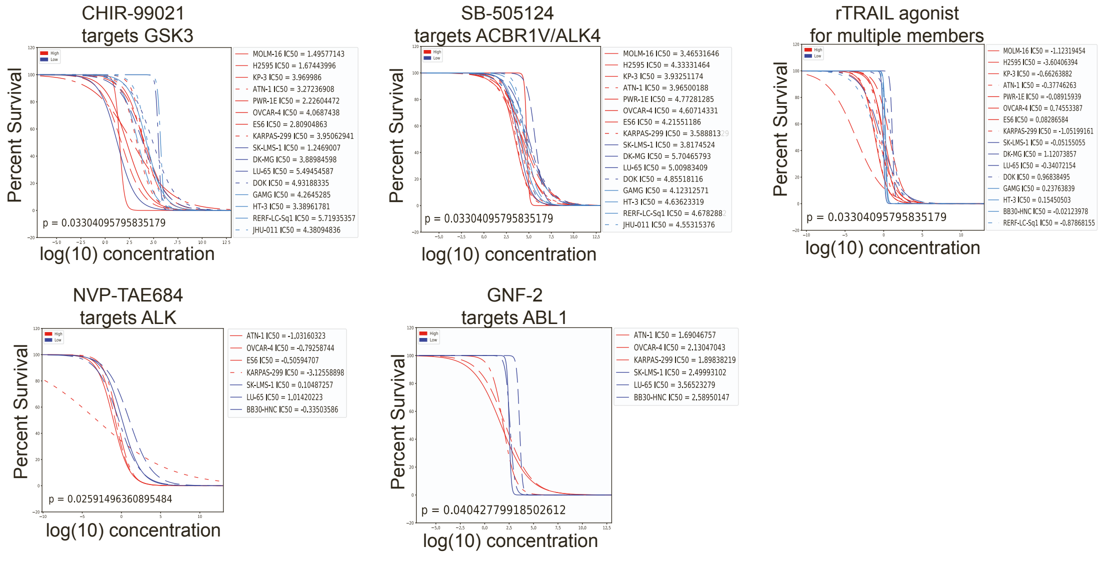
CHIR-99021
targets GSK3
Percent Survival
log(10) concentration
p = 0.033040957795835179
High
Low
MOLM-16 IC50 = 1.49577143
H2595 IC50 = 1.67443996
KP-3 IC50 = 3.969986
ATN-1 IC50 = 3.27236908
PWR-1E IC50 = 2.22604472
OVCAR-4 IC50 = 4.0687438
ES6 IC50 = 2.80904863
KARPAS-299 IC50 = 3.95062941
SK-LMS-1 IC50 = 1.2469007
DK-MG IC50 = 3.88984598
LU-65 IC50 = 5.49454587
DOK IC50 = 4.93188335
GAMG IC50 = 4.2645285
HT-3 IC50 = 3.38961781
RERF-LC-Sq1 IC50 = 5.71935357
JHU-011 IC50 = 4.38094836
SB-505124
targets ACBR1V/ALK4
Percent Survival
log(10) concentration
p = 0.033040957795835179
MOLM-16 IC50 = 3.46531646
H2595 IC50 = 4.33331464
KP-3 IC50 = 3.93251174
ATN-1 IC50 = 3.96500188
PWR-1E IC50 = 4.77281285
OVCAR-4 IC50 = 4.60714331
ES6 IC50 = 4.21551186
KARPAS-299 IC50 = 3.588813
SK-LMS-1 IC50 = 3.8174524
DK-MG IC50 = 5.70465793
LU-65 IC50 = 5.00983409
DOK IC50 = 4.85518116
GAMG IC50 = 4.12312571
HT-3 IC50 = 4.63623319
RERF-LC-Sq1 IC50 = 4.678288
JHU-011 IC50 = 4.55315376
rTRAIL agonist
for multiple members
Percent Survival
log(10) concentration
p = 0.033040957795835179
MOLM-16 IC50 = -1.12319454
H2595 IC50 = -3.60406394
KP-3 IC50 = -0.66263882
ATN-1 IC50 = -0.37746263
PWR-1E IC50 = -0.08915939
OVCAR-4 IC50 = 0.74553387
ES6 IC50 = 0.08286584
KARPAS-299 IC50 = -1.05199161
SK-LMS-1 IC50 = -0.05155055
DK-MG IC50 = 1.12073857
LU-65 IC50 = -0.34072154
DOK IC50 = 0.96838495
GAMG IC50 = 0.23763839
HT-3 IC50 = 0.15450503
BB30-HNC IC50 = -0.02123978
RERF-LC-Sq1 IC50 = -0.87868155
NVP-TAE684
targets ALK
Percent Survival
log(10) concentration
p = 0.025914963608954484
ATN-1 IC50 = -1.03160323
OVCAR-4 IC50 = -0.79258744
ES6 IC50 = -0.50594707
KARPAS-299 IC50 = -3.12558898
SK-LMS-1 IC50 = 0.10487257
LU-65 IC50 = 1.01420223
BB30-HNC IC50 = -0.33503586
GNF-2
targets ABL1
Percent Survival
log(10) concentration
p = 0.040427779918502612
ATN-1 IC50 = 1.69046757
OVCAR-4 IC50 = 2.13047043
KARPAS-299 IC50 = 1.89838219
SK-LMS-1 IC50 = 2.49993102
LU-65 IC50 = 3.56523279
BB30-HNC IC50 = 2.58950147

Figure S2. Enrichment analyses of the genome-wide, loss-of-function data, related to Figure 1. A. Cytoscape network of all 960 SDL hits identified by genome-wide shRNA screens. Each node represents a hit from the screen. The genes are color coded based on their gene ontology. The blue edges represent previously published interactions downloaded from the STRING database showing crosstalk among the SDL hits. **B.** IC50 curves of drug inhibitors of some of the SDL hits identified in the screen. The red sigmoidal curve represents the IC50 curve for PLK1-overexpressing cell lines, and the blue sigmoidal curves represent the IC50 curves for cell lines with low PLK1 expression.

Fig S3

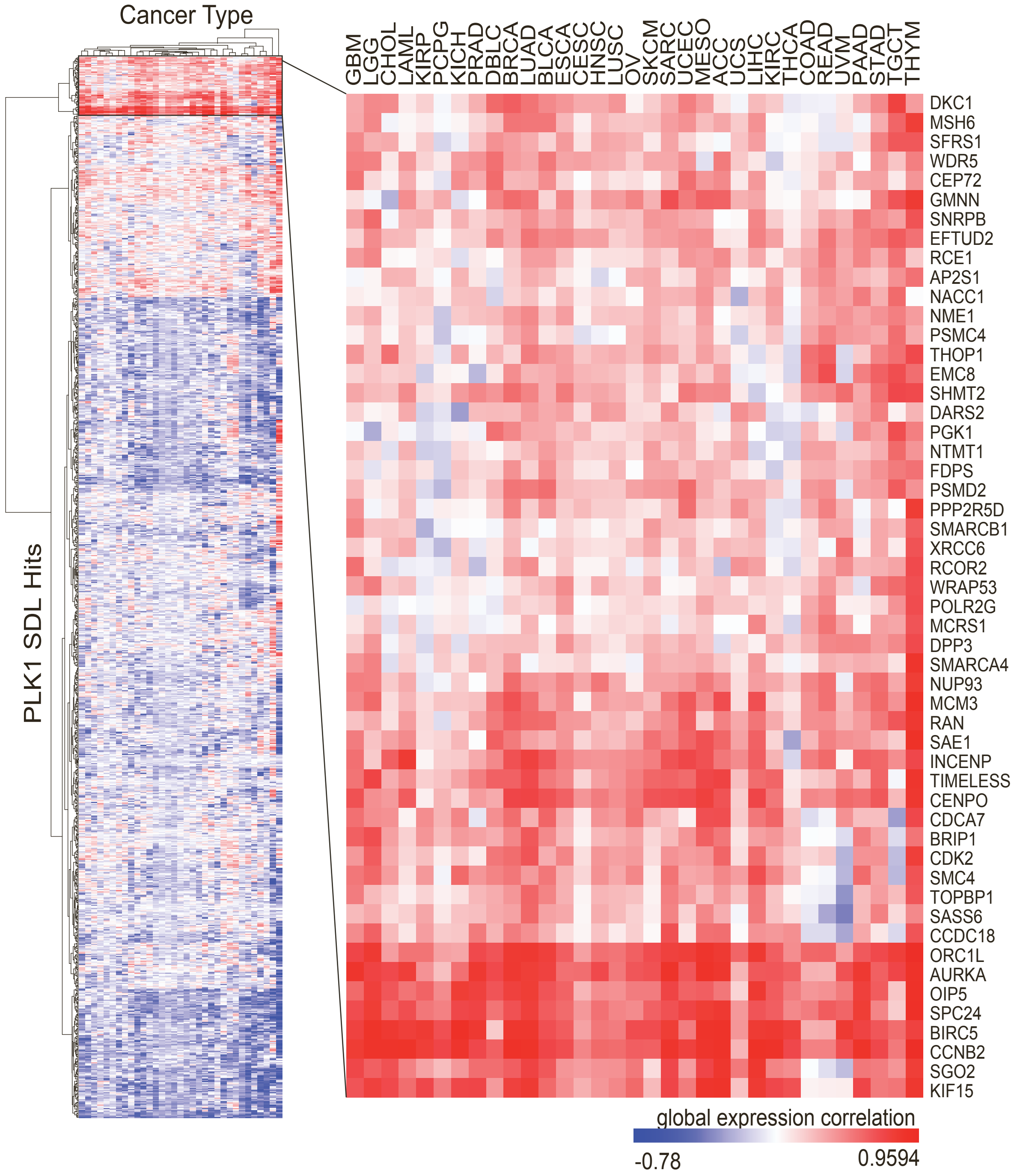
Cancer Type
PLK1 SDL Hits
GBM
LGG
CHOL
LAML
KIRP
PCPG
KICH
PRAD
DBLC
BRCA
LUAD
BLCA
ESCA
CESC
HNSC
LUSC
OV
SKCM
SARC
UCEC
MESO
ACC
UCS
LIHC
KIRC
THCA
COAD
READ
UVM
PAAD
STAD
TGCT
THYM
DKC1
MSH6
SFRS1
WDR5
CEP72
GMNN
SNRPB
EFTUD2
RCE1
AP2S1
NACC1
NME1
PSMC4
THOP1
EMC8
SHMT2
DARS2
PGK1
NTMT1
FDPS
PSMD2
PPP2R5D
SMARCB1
XRCC6
RCOR2
WRAP53
POLR2G
MCRS1
DPP3
SMARCA4
NUP93
MCM3
RAN
SAE1
INCENP
TIMELESS
CENPO
CDCA7
BRIP1
CDK2
SMC4
TOPBP1
SASS6
CCDC18
ORC1L
AURKA
OIP5
SPC24
BIRC5
CCNB2
SGO2
KIF15
global expression correlation
-0.78
0.9594

Figure S3. Expression-based prioritization of SDL hits from the genome-wide, loss-of-function data, related to Figure 1. A. Hierarchical clustering of the correlation between the expression of PLK1 and the expression of each SDL hit across 33 cancer types from The Cancer Genome Atlas (TCGA) patient data obtained from Genomic Data Commons (GDC).

Fig S4

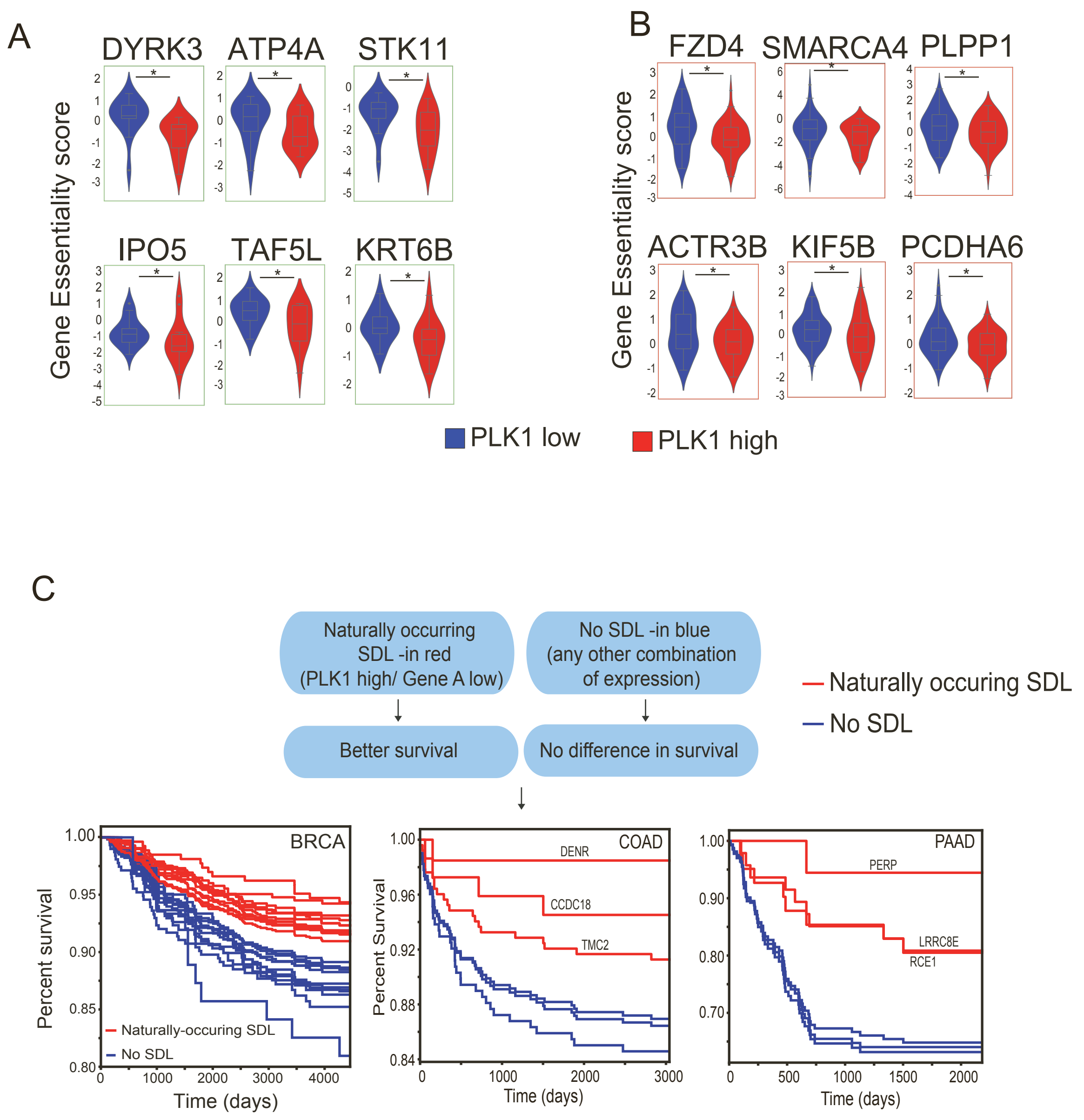

A
DYRK3
ATP4A
STK11
IPO5
TAF5L
KRT6B
Gene Essentiality score
B
FZD4
SMARCA4
PLPP1
ACTR3B
KIF5B
PCDHA6
Gene Essentiality score
PLK1 low
PLK1 high
C
Naturally occurring SDL -in red (PLK1 high/ Gene A low)
No SDL -in blue (any other combination of expression)
Better survival
No difference in survival
Naturally occuring SDL
No SDL
BRCA
Percent survival
Naturally-occuring SDL
No SDL
Time (days)
COAD
DENR
CCDC18
TMC2
Percent Survival
Time (days)
PAAD
PERP
LRRC8E
RCE1
Time (days)

Figure S4. DepMap and patient survival-based prioritization of SDL hits from the genome-wide, loss-of-function data, related to Figure 1. A. and B. Violin plots of the difference in essentiality scores for PLK1 SDL hits in different cell lines grouped by low versus high PLK1 expression. Essentiality scores derived from published shRNA screening data (Marcotte *et al*., highlighted in green) and the Project Achilles database (https://depmap.org/portal/achilles/) (highlighted in orange). The p value significance was calculated using the non-parametric Wilcoxon rank sum test. **D.** Schematic of the identification of naturally occurring SDL interactions in patients with breast cancer (BRCA) using Kaplan–Meier survival curves. Log rank p values were computed to calculate significance. Few representative examples of Kaplan–Meier plots for colon and pancreatic cancer patients displaying natural SDL expression patterns are presented**.**

Fig S5

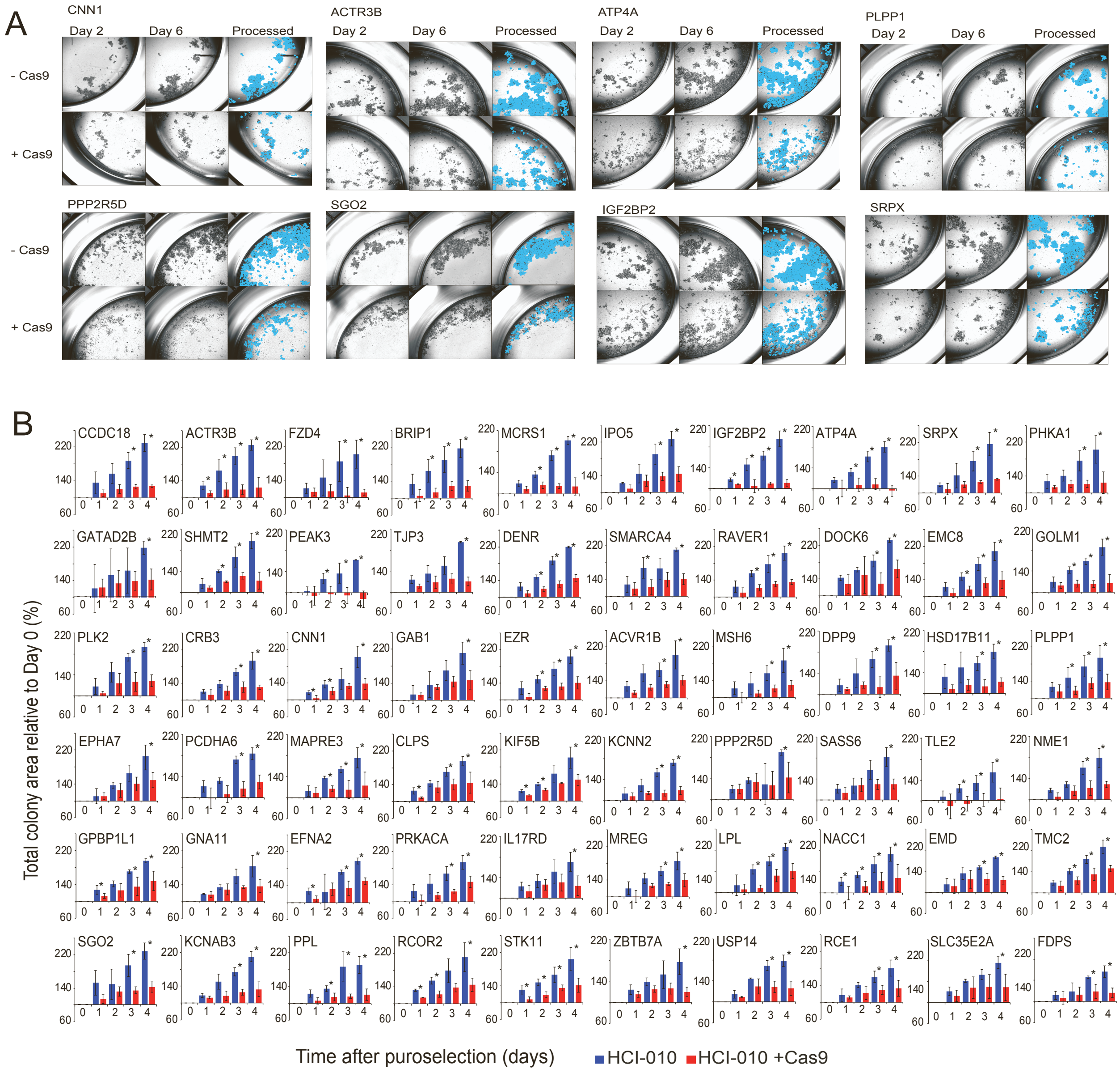
A
CNN1
ACTR3B
ATP4A
PLPP1
Day 2
Day 6
Processed
- Cas9
+ Cas9
PPP2R5D
SGO2
IGF2BP2
SRPX
B
Total colony area relative to Day 0 (%)
CCDC18
ACTR3B
FZD4
BRIP1
MCRS1
IPO5
IGF2BP2
ATP4A
SRPX
PHKA1
GATAD2B
SHMT2
PEAK3
TJP3
DENR
SMARCA4
RAVER1
DOCK6
EMC8
GOLM1
PLK2
CRB3
CNN1
GAB1
EZR
ACVR1B
MSH6
DPP9
HSD17B11
PLPP1
EPHA7
PCDHA6
MAPRE3
CLPS
KIF5B
KCNN2
PPP2R5D
SASS6
TLE2
NME1
GPBP1L1
GNA11
EFNA2
PRKACA
IL17RD
MREG
LPL
NACC1
EMD
TMC2
SGO2
KCNAB3
PPL
RCOR2
STK11
ZBTB7A
USP14
RCE1
SLC35E2A
FDPS
Time after puroselection (days)
HCI-010
HCI-010 +Cas9

Figure S5. A. Arrayed CRISPR-based validation of prioritized SDL hits from the genome-wide, loss-of-function data, related to Figure 1. Representative images acquired using automated imaging over time for different PLK1-SDL candidate knockouts in $Cas9^-$ and $Cas9^+$ HCI-010 cells. The MetaXpress object masking overlay is shown in blue for the day 6 images. **B.** Bar graph quantification of the imaging analysis over the course of 4 days. n = 3, * p value < 0.05, Student's t test.

Fig S6

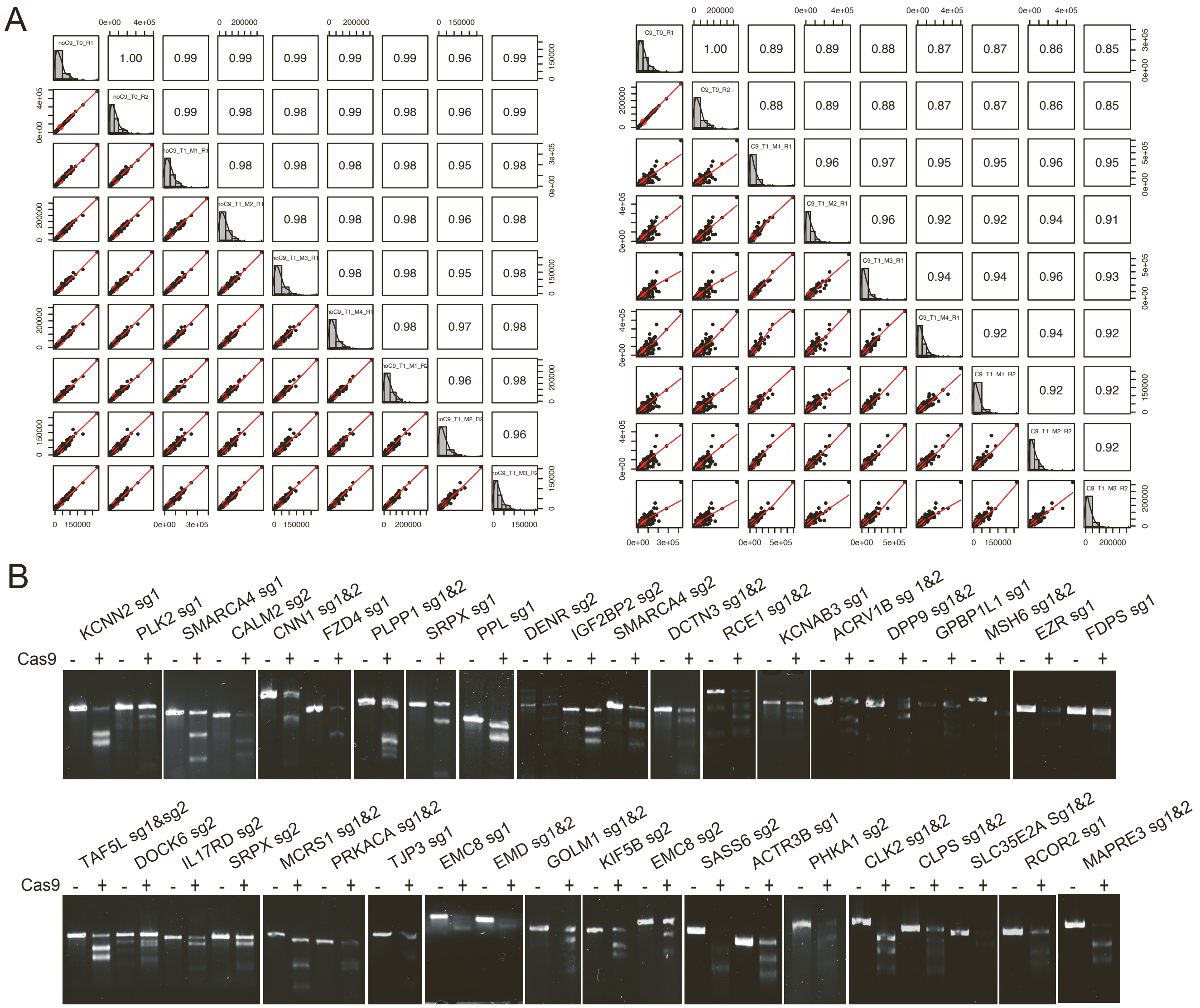
A
B
KCNN2 sg1
PLK2 sg1
SMARCA4 sg1
CALM2 sg2
CNN1 sg1&2
FZD4 sg1
PLPP1 sg1&2
SRPX sg1
PPL sg1
DENR sg2
IGF2BP2 sg2
SMARCA4 sg2
DCTN3 sg1&2
RCE1 sg1&2
KCNAB3 sg1
ACRV1B sg 1&2
DPP9 sg1&2
GPBP1L1 sg1
MSH6 sg1&2
EZR sg1
FDPS sg1
Cas9
TAF5L sg1&sg2
DOCK6 sg2
IL17RD sg2
SRPX sg2
MCRS1 sg1&2
PRKACA sg1&2
TJP3 sg1
EMC8 sg1
EMD sg1&2
GOLM1 sg1&2
KIF5B sg2
EMC8 sg2
SASS6 sg2
ACTR3B sg1
PHKA1 sg2
CLK2 sg1&2
CLPS sg1&2
SLC35E2A Sg1&2
RCOR2 sg1
MAPRE3 sg1&2
Cas9

Figure S6. Screening quality of in vivo CRISPR screening and genome editing efficiency of prioritized SDL hits, related to Figure 1. A. Correlation plots between replicates of the *in vivo* pooled CRISPR screen. The left panel shows the Cas9-negative samples, and the right panel shows the Cas9-positive samples. **B.** Representative cleavage assay confirming the individual knockouts.

Fig S7

A

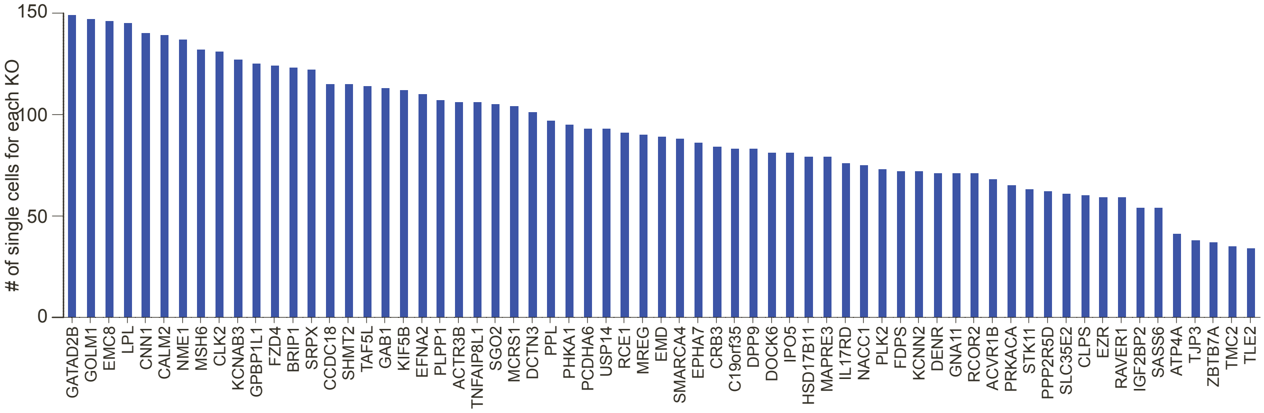

B

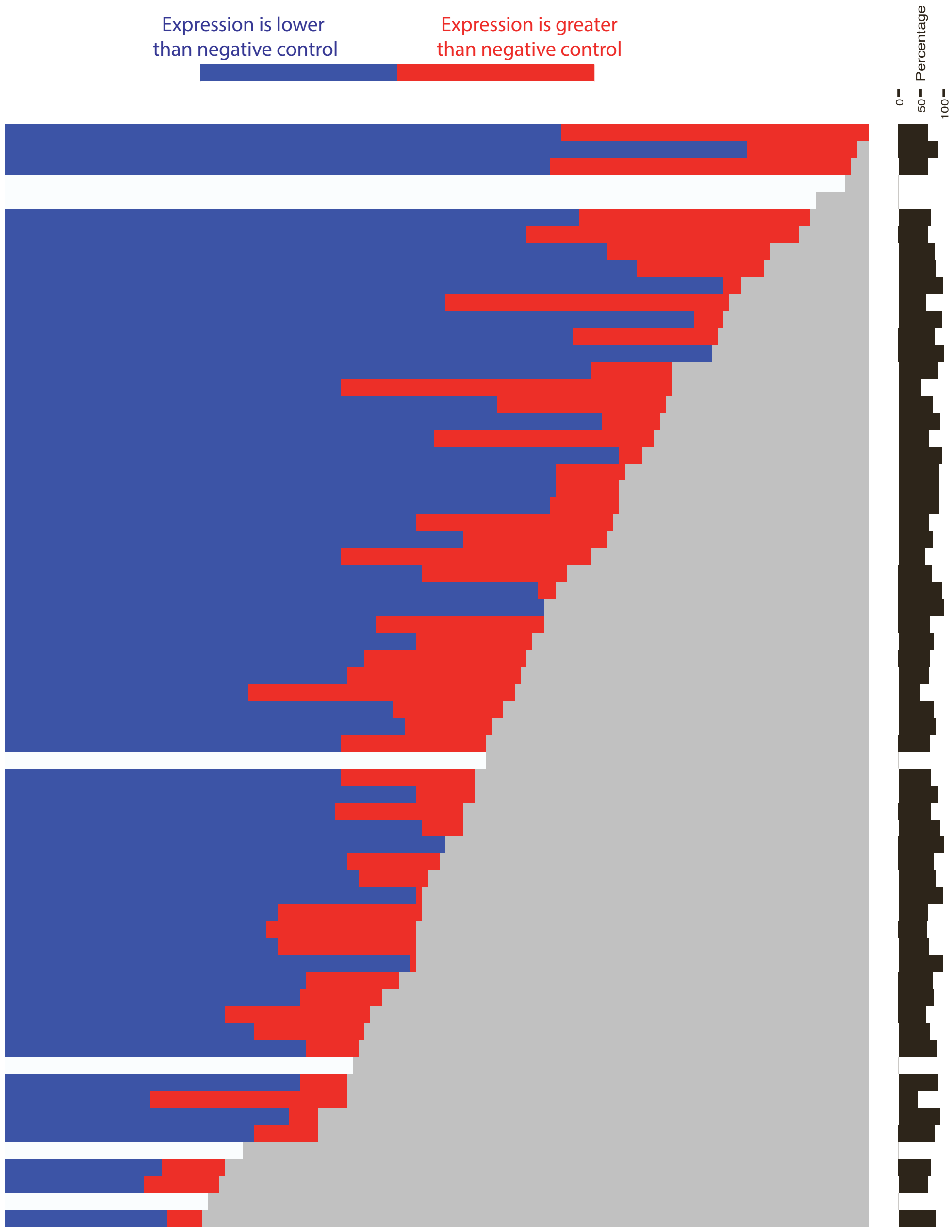

GeneSymbol	Count	Percentage
GATAD2B	149	64.43
GOLM1	147	87.07
EMC8	146	64.38
LPL	145	0
CNN1	140	0
CALM2	139	71.22
NME1	137	65.69
MSH6	132	78.79
CLK2	131	83.21
KCNAB3	127	97.64
GPBP1L1	125	60.8
FZD4	124	95.97
BRIP1	123	79.67
SRPX	122	100
CCDC18	115	87.83
SHMT2	115	50.43
TAF5L	114	74.56
GAB1	113	91.15
KIF5B	112	66.07
EFNA2	110	96.36
PPAP2A	107	88.79
ACTR3B	106	89.62
TNFAIP8L1	106	88.68
SGOL2	105	67.62
MCRS1	104	75.96
DCTN3	101	57.43
PPL	97	74.23
PHKA1	95	96.84
PCDHA6	93	100
USP14	93	68.82
RCE1	91	78.02
MREG	90	68.89
EMD	89	66.29
SMARCA4	88	47.73
EPHA7	86	77.91
CRB3	84	82.14
DPP9	83	69.88
C19orf35	83	0
IPO5	81	71.6
DOCK6	81	87.65
MAPRE3	79	72.15
HSD17B11	79	91.14
IL17RD	76	100
NACC1	75	78.67
PLK2	73	83.56
KCNN2	72	98.61
FDPS	72	65.28
GNA11	71	63.38
DENR	71	66.2
RCOR2	71	98.59
ACVR1B	68	76.47
PRKACA	65	78.46
STK11	63	60.32
PPP2R5D	62	69.35
SLC35E2	61	85.25
CLPS	60	0
EZR	59	86.44
RAVER1	59	42.37
IGF2BP2	54	90.74
SASS6	54	79.63
ATP4A	41	0
TJP3	38	71.05
ZBTB7A	37	64.86
TMC2	35	0
TLE2	34	82.35

Figure S7. Analyses of direct-capture, perturb-seq of prioritized SDL hits, related to Figure 2. A Bar graph showing the number of cells with each individual knockout analyzed via single-cell direct capture Perturb-seq. **B.** Knockout efficiency in the single-cell CRISPR screen was confirmed by comparing the expression of the corresponding target gene between cells from negative controls and knockouts. The percentage of knockout efficiency for each target is presented in addition to the heatmap.

Fig S8

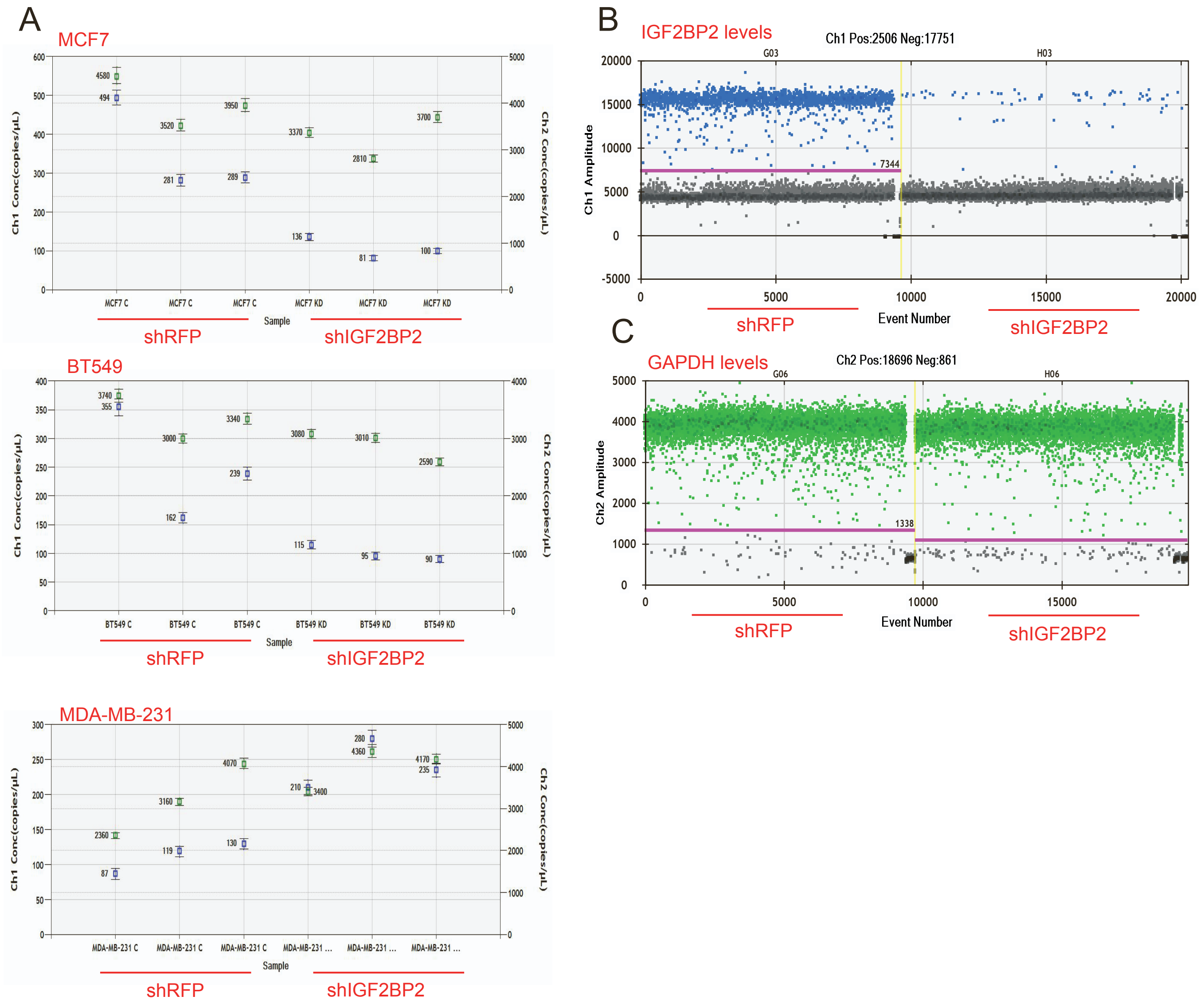

A
MCF7
Ch1 Conc(copies/µL)
Ch2 Conc(copies/µL)
4580
494
3520
281
3950
289
3370
136
2810
81
3700
100
MCF7 C
MCF7 KD
Sample
shRFP
shIGF2BP2
BT549
3740
355
3000
162
3340
239
3080
115
3010
95
2590
90
BT549 C
BT549 KD
Sample
shRFP
shIGF2BP2
MDA-MB-231
2360
87
3160
119
4070
130
210
3400
280
4360
4170
235
MDA-MB-231 C
MDA-MB-231 ...
Sample
shRFP
shIGF2BP2
B
IGF2BP2 levels
Ch1 Pos:2506 Neg:17751
G03
H03
Ch1 Amplitude
7344
Event Number
shRFP
shIGF2BP2
C
GAPDH levels
Ch2 Pos:18696 Neg:861
G06
H06
Ch2 Amplitude
1338
Event Number
shRFP
shIGF2BP2

Figure S8. Raw data output from digital droplet PCR, related to Figure 3. A. Absolute quantification (concentration in copies/µL) of GAPDH (VIC-labeled, green) and PLK1 (FAM-labeled, blue) in 3 cell lines, the shRFP control and shIGF2BP2 knockdown samples. **B.** Representative 1D plot showing positive (blue) and negative (gray) droplets based on the IGF2BP2 probe (FAM-labeled) in shRFP control and shIGF2BP2 knockdown cell line samples. **C.** Representative 1D plot showing positive (green) and negative (gray) droplets based on the use of a GAPDH probe (VIC-labeled) in shRFP control and shIGF2BP2 knockdown cell line samples.

Fig S9

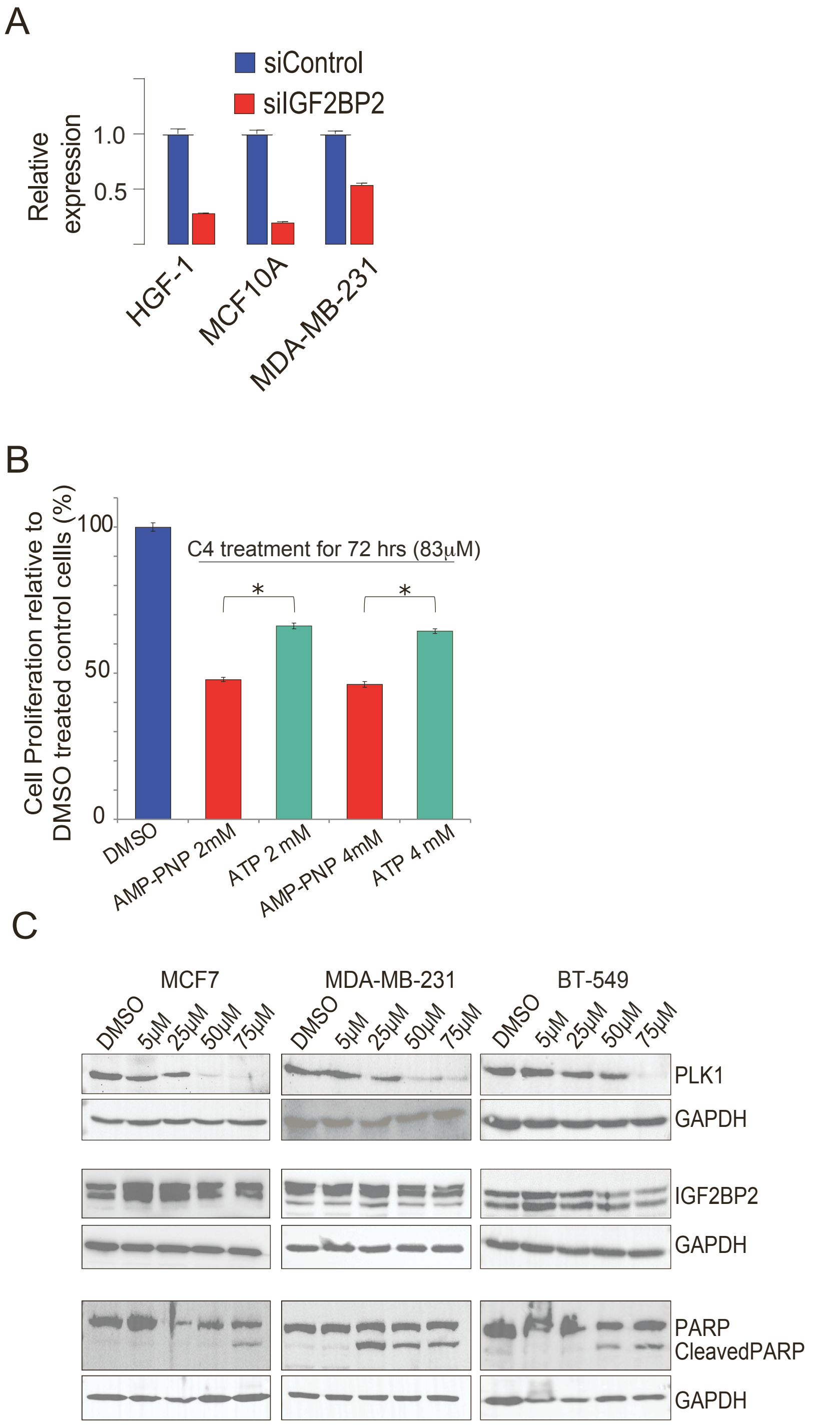
A
siControl
siIGF2BP2
Relative expression
1.0
0.5
HGF-1
MCF10A
MDA-MB-231
B
Cell Proliferation relative to DMSO treated control cells (%)
100
50
0
C4 treatment for 72 hrs (83μM)
*
*
DMSO
AMP-PNP 2mM
ATP 2 mM
AMP-PNP 4mM
ATP 4 mM
C
MCF7
MDA-MB-231
BT-549
DMSO
5μM
25μM
50μM
75μM
PLK1
GAPDH
IGF2BP2
GAPDH
PARP
CleavedPARP
GAPDH

Figure S9. Effects of Pharmacological inhibition of IGF2BP2, related to Figure 5. A. RT-qPCR results following the siRNA knockdown of IGF2BP2 in both non-malignant and malignant (MDA-MB-231) cells used in this study is presented. **B.** CCK8 assay of cell viability after exposure of MDA-MB-231 cells to 83 μM of the IGF2BP2 inhibitor C4 for 72 hours, in the absence or presence of 2 or 4 mM exogenous ATP or the non-hydrolyzable ATP analog AMP-PNP. All data are expressed as a function of vehicle-alone treated MDA-MB-231 cells. **C.** Western blot showing the levels of PLK1, IGF2BP2 and PARP after treatment with an IGF2BP2 inhibitor (C4) in MCF7, MDA-MB-231 and BT-549 cells at doses ranging from 5 to 75 μM for 72 hours. GAPDH was used as a loading control.
